# Interpretable detection-based image classification for differential diagnosis of vaginal discharge using smartphone-assisted microscopy

**DOI:** 10.3389/frai.2026.1985517

**Published:** 2026-10-15

**Authors:** Siriwoot Sookkhee, Marut Wongtapin, Trin Phuriyaphan, Kornkaran Tonpiriyaphong, Nitan Wachirapaet, Surapee Wasanapitanont, Jidapa Joysa, Monsicha Pongpom, Narin Lawan, Phit Upaphong, Tanaporn Wangsanut

**Affiliations:** 1 Department of Microbiology, Faculty of Medicine, Chiang Mai University, Chiang Mai, Thailand; 2 Department of Chemistry, Faculty of Science, Chiang Mai University, Chiang Mai, Thailand; 3 Department of Ophthalmology, Faculty of Medicine, Chiang Mai University, Chiang Mai, Thailand

**Keywords:** artificial intelligence, digital health, medical image analysis, sexually transmitted infections, smartphone AI, vaginal discharge, vaginal pathogen

## Abstract

**Background:**

The vaginal discharge is a common clinical presentation associated with diverse infectious and microbiota-related conditions. Existing artificial intelligence (AI)-based diagnostic approaches have predominantly focused on detection of individual pathogens and have rarely integrated microbiota-associated conditions within a diagnostic framework. We developed an explainable deep learning framework for automated analysis of Gram-stained vaginal smear images acquired using handheld smartphones or tablets positioned over a conventional light microscope eyepiece.

**Methods:**

The framework integrates object detection with image-level classification, termed detection-based image classification, to identify diagnostically relevant microscopic structures without requiring exhaustive enumeration of all objects. Five diagnostic categories included vulvovaginal candidiasis (VVC), bacterial vaginosis (BV), trichomoniasis (TV), gonococcal urethritis/cervicitis (GU), and healthy vaginal condition (HL). YOLOv26, RT-DETR and EfficientDet0 were independently trained and evaluated using 1,966 microscopic images comprising 11,069 annotated diagnostic objects. Model explainability and computational benchmarking were assessed. The localization quality was independently evaluated by eight clinicians and microbiologists.

**Results:**

Class-specific analysis demonstrated that TV and GU consistently achieved the highest detection performance across all models (mAP50 = 0.622–0.752), whereas RT-DETR demonstrated superior performance for VVC, BV, and HL. Expert evaluation showed favorable localization, with Good/Excellent ratings in 84.8 and 85.1% of valid evaluations for YOLOv26 and RT-DETR, respectively, with no significant difference between models (OR = 0.97; 95% CI, 0.58–1.61; *p* = 0.895).

**Conclusion:**

This study presents an accurate and clinically interpretable framework that integrates microbiota-associated conditions and sexually transmitted infections for differential diagnosis of vaginal discharge using conventional light microscopy. By linking image-level predictions to localized diagnostic morphology, the framework provides human-verifiable AI evidence as a proof-of-concept prototype. While promising for low-cost implementation, rigorous prospective multi-center validation is required before broad clinical deployment or autonomous diagnosis.

## 1 Introduction

Pathogenic vaginal discharge (vaginal leukorrhea) is among the most common gynecological complaints encountered in primary care and sexual health clinics. Common clinical manifestations include pruritus, dyspareunia, vaginal discomfort, abnormal vaginal discharge, and malodor, although the presentation may vary according to the underlying cause (Shroff, 2023). The condition occurs most frequently in women of reproductive age and is commonly associated with hormonal changes, sexual activity, and genital infections. The differential diagnosis includes vulvovaginal candidiasis (VVC), bacterial vaginosis (BV), and sexually transmitted infections such as trichomoniasis (TV) and gonococcal cervicitis and/or urethritis (GU). Distinguishing these conditions from physiologic vaginal discharge associated with a healthy vaginal microbiota is essential to avoid unnecessary treatment and ensure appropriate clinical management. The etiologies of these conditions differ substantially. VVC and BV are associated with alterations of the vaginal microbial ecosystem; while VVC results from overgrowth of *Candida albicans,* BV is characterized by depletion of *Lactobacillus*-dominated microbiota and proliferation of anaerobic bacteria, including *Gardnerella vaginalis*, *Atopobium vaginae*, *Mobiluncus* spp., and *Prevotella* spp. (Gaziano et al., 2023; Sadeghpour Heravi, 2024). In contrast, TV and GU are caused by the sexually transmitted pathogens *Trichomonas vaginalis* and *Neisseria gonorrhoeae*, respectively (Rowley et al., 2019). Because these infections often present with overlapping clinical manifestations, laboratory confirmation is frequently required for accurate etiological diagnosis. Correct diagnosis guides appropriate therapy, reduces unnecessary antimicrobial use, and minimizes treatment failure and other adverse consequences of inappropriate management.

Among the available diagnostic methods, microscopic examination of Gram-stained vaginal specimens remains a rapid, inexpensive, and widely accessible diagnostic method, particularly in resource-limited settings (Thomson, 2016; Workowski et al., 2021). Pseudohyphae represent the invasive morphological form of *Candida* spp. and are indicative of VVC (Valentine et al., 2025). The detection of clue cells, vaginal epithelial cells extensively covered with adherent *G. vaginalis* organisms over their entire cell surface, is a hallmark microscopic feature of BV (Swidsinski et al., 2022; Muzny et al., 2023). The presence of motile flagellated *Trichomonas* trophozoites is diagnostic of TV (Harrington and Williams, 1999; Van Der Pol, 2016; Ortega-Ojeda et al., 2026). GU is characterized by the presence of intracellular Gram-negative diplococci within leukocytes (Rietmeijer and Mettenbrink, 2012). In contrast, healthy vaginal samples are characterized by epithelial cells colonized predominantly by *Lactobacillus* spp. (Valentine et al., 2025). Despite its diagnostic value, microscopy remains under-utilized in routine clinical practice (Hillier et al., 2021). Hillier et al. reported that microscopy was performed in only 17.4% of visits among women presenting with vulvovaginal symptoms. Even when available, accurate microscopic interpretation requires experienced personnel and is subject to inter-observer variability. In addition, conventional microscopy relies on laboratory infrastructure and trained personnel, resources that are often available only in hospitals or specialized medical centers. Consequently, access to microscopy-based diagnosis may be restricted in low-resource or underserved settings, delaying accurate diagnosis and appropriate treatment. This is particularly important during pregnancy, in which untreated or inadequately treated vaginal infections have been associated with adverse maternal and fetal outcomes (Li et al., 2022; Duarte et al., 2024; Yao et al., 2025).

Recent advances in Artificial Intelligence (AI), particularly deep learning-based approaches, have shown considerable promise in the automated analysis of microscopic images, enabling accurate, robust, and scalable image interpretation across diverse biomedical applications. However, most previous studies have focused on detecting a single pathogen or disease entity, limiting their applicability to routine clinical practice where multiple etiologies must be considered simultaneously (Ortega-Ojeda et al., 2026; Wang et al., 2021; Nguyen et al., 2025; Wang et al., 2025; Pongpom et al., 2026). Furthermore, few studies have incorporated vaginal microbiota-associated conditions, such as *Lactobacillus*-dominated flora into a unified deep learning framework alongside infectious pathogens (Watanabe et al., 2025). Second, most existing approaches propose microscopic diagnosis either as object detection, in which individual structures are localized and classified, or as image classification, in which an image is assigned a specimen-level label without explicit localization. These approaches present complementary limitations for diagnostic microscopy. Exhaustive object detection may penalize clinically acceptable predictions when only a representative diagnostic structure is required for diagnosis, whereas conventional image classification provides limited visual evidence for how a prediction was generated. In addition, most AI-assisted microscopy systems have been developed using digitized microscopy platforms, whereas routine microscopic examinations in many clinical laboratories are still performed using conventional light microscopes without integrated digital image acquisition. These limitations hinder the translation of AI-assisted microscopy into routine clinical practice.

To address these limitations, we developed an explainable deep learning framework for the differential diagnosis of common causes of vaginal discharge using smartphone-acquired images from a conventional light microscope. Building on our previous work demonstrating automated detection of fungal elements associated with VVC from handheld mobile device-acquired microscopic images (Pongpom et al., 2026), we extended the framework to simultaneously identify VVC, BV, TV, GU, and healthy vaginal specimens, enabling comprehensive multi-class diagnosis. We compared three object detection architectures, YOLOv26, RT-DETR, and EfficientDet-D0, evaluating diagnostic performance, model explainability, inference efficiency, and computational complexity to inform their potential use in point-of-care microscopy. To better align with routine microscopic interpretation, we further developed a detection-based image classification framework that generates specimen-level diagnoses from detected diagnostic features. To our knowledge, this is the first study to develop an interpretable deep learning framework for the simultaneous microscopic detection of the major causes of abnormal vaginal discharge, encompassing both microbiota-associated conditions and sexually transmitted pathogens, using smartphone-acquired images from a conventional light microscope.

## 2 Results

### 2.1 Dataset composition and object detection model evaluation

The dataset comprised Gram-stained vaginal discharge microscopy images obtained from two annotation sources. The first dataset contained 1,507 images with bounding-box annotations for four diagnostic categories, TV, GU, BV, and HL. The second dataset consisted of 459 images annotated with segmentations for VVC; these annotations were subsequently converted into bounding boxes for model training and evaluation. Collectively, the combined dataset included 1,966 microscopic images comprising 11,069 annotated diagnostic objects (Table 1).

**Table 1:** Dataset composition.

Split	Images	Box instance				
		TV	GU	BV	HL	VVC
Train	1,003	107	553	1,089	1,782	643
Validation	449	89	389	635	1,874	603
Test	514	80	281	482	1,195	1,267
Total	1,966	276	1,223	2,206	4,851	2,513

BV, bacterial vaginosis; GU, gonococcal cervicitis and/or urethritis; HL, healthy vaginal condition; TV, trichomoniasis; VVC, vulvovaginal candidiasis.

Hyperparameter optimization was conducted for each object detection architecture using a validation dataset, comprising 449 images and 3,590 annotated instances distributed across five diagnostic categories (TV, GU, BV, HL, and VVC) (Supplementary Table S1). Final model performance was subsequently evaluated using an independent test dataset comprising 514 images and 3,305 annotated instances representing the same diagnostic categories. Among the evaluated models, RT-DETR achieved the highest overall detection performance on the independent test dataset (mAP50 = 0.382), followed by YOLOv26 (mAP50 = 0.357), whereas EfficientDet-D0 exhibited substantially lower performance (mAP50 = 0.116) (Table 2). Although RT-DETR attained the highest mAP50, YOLOv26 remained competitive and efficiently localized diagnostically relevant microbial targets (Figures 1–3). Compared with YOLOv26, RT-DETR achieved higher recall (0.458 vs. 0.410) while maintaining comparable precision, indicating greater sensitivity (recall) for object detection, albeit with a modest increase in false-positive detections.

**Table 2:** Overall test set performance.

Model	Precision	Recall	mAP50	mAP50-95
YOLOv26 v2	0.399	0.410	0.357	0.174
RT-DETR v2	0.406	0.458	0.382	0.196
EfficientDet-D0	–	–	0.116	0.028

mAP50, mean average precision at an intersection over union (IoU) threshold of 0.50; mAP50–95, mean average precision averaged across IoU thresholds from 0.50 to 0.95.

**Figure 1:**
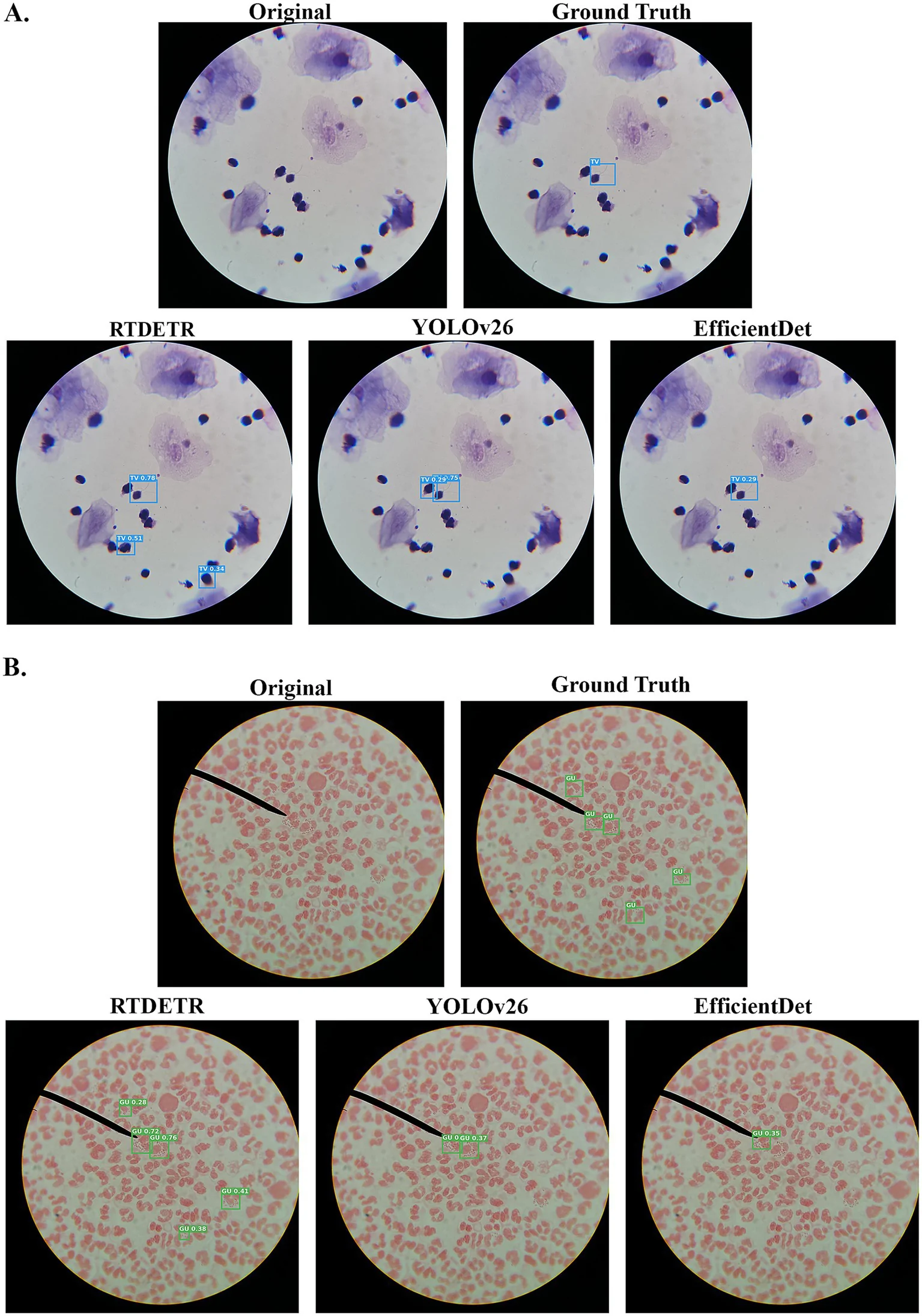
Visual comparison of object detection performance across model architectures. Representative predictions from YOLOv26, RT-DETR, and EfficientDet-D0 on Gram-stained microscopic images are shown. Examples from the trichomoniasis (TV) **(A)** and gonococcal cervicitis and/or urethritis (GU) **(B)** classes are presented, as these classes achieved the highest detection performance in the test set. Predicted bounding boxes and class labels illustrate each model’s ability to localize diagnostically relevant microscopic features. Numbers above bounding boxes indicate the model’s confidence for each detection.

**Figure 2:**
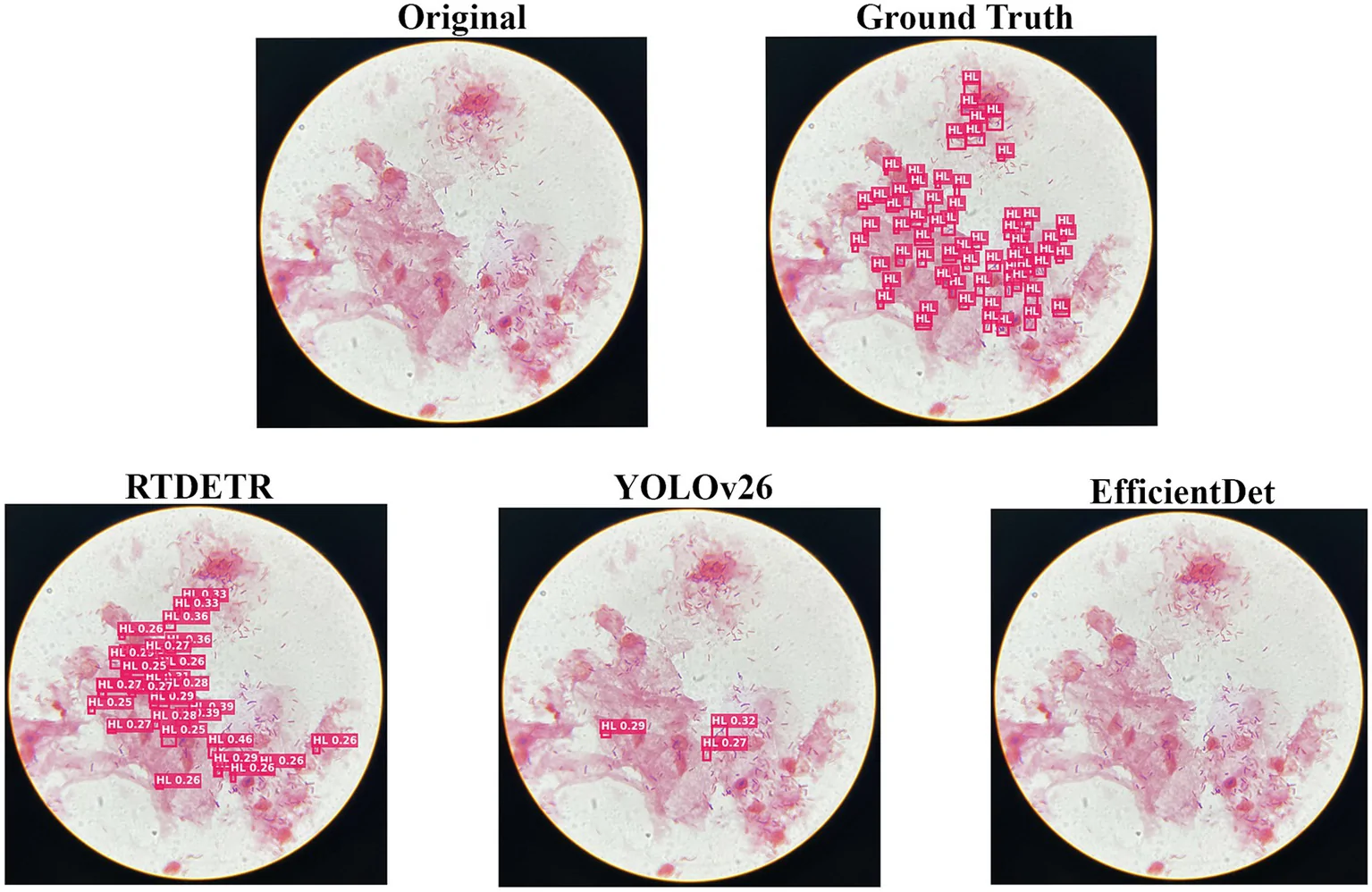
Visual comparison of object detection performance across model architectures. Representative predictions from YOLOv26, RT-DETR, and EfficientDet-D0 on Gram-stained microscopic images are shown. Examples from the healthy vaginal condition (HL) class are presented, as this class showed challenging detection performance in the test set, particularly with respect to accurate counting. Predicted bounding boxes and class labels illustrate each model’s ability to localize diagnostically relevant microscopic features. Numbers above bounding boxes indicate the model’s confidence for each detection.

**Figure 3:**
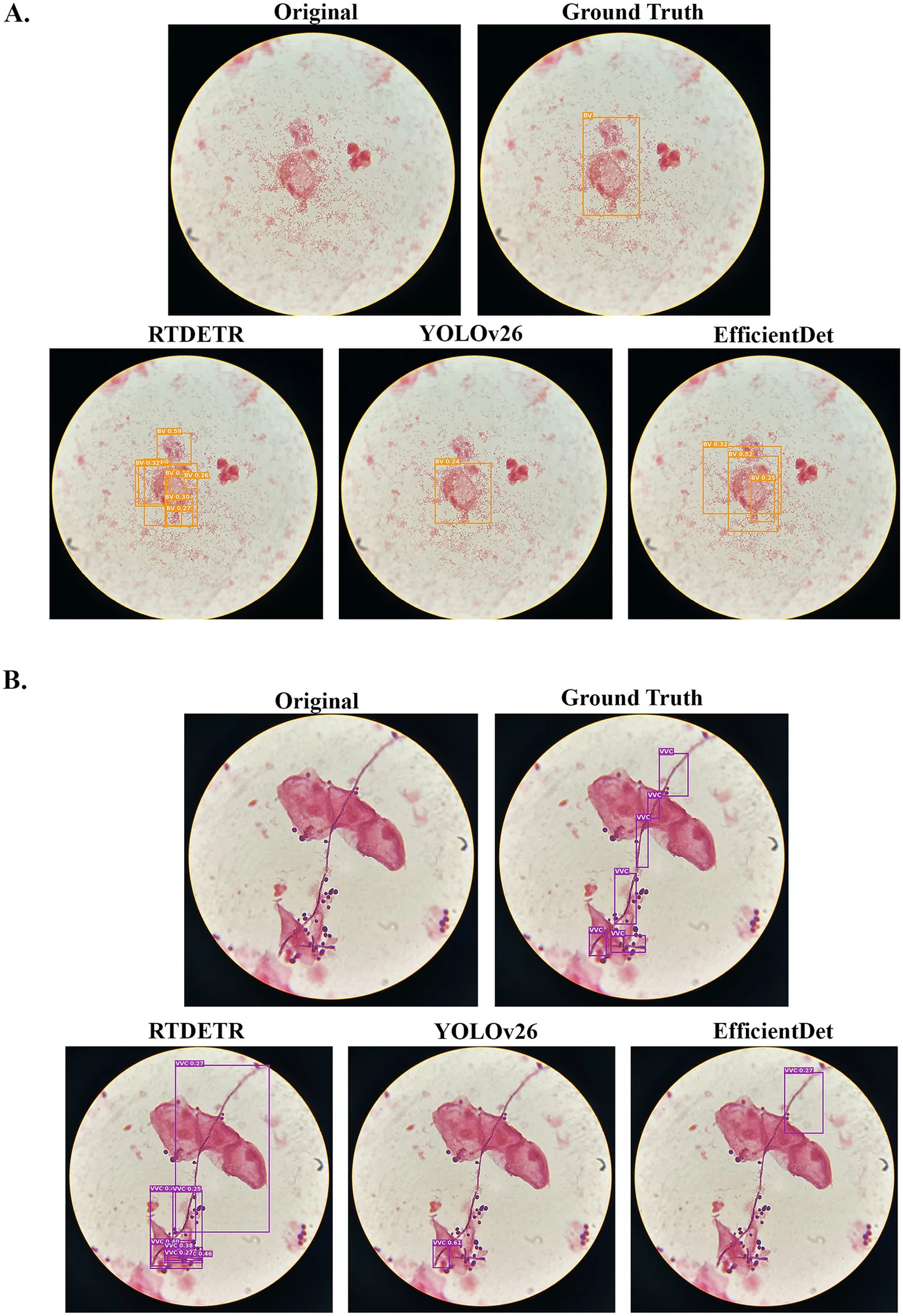
Visual comparison of object detection performance across model architectures. Representative predictions from YOLOv26, RT-DETR, and EfficientDet-D0 on Gram-stained microscopic images are shown. Examples from the bacterial vaginosis (BV) **(A)** and vulvovaginal candidiasis (VVC) **(B)** classes are presented, as these two classes showed challenging detection performance on the test set due to their irregular morphologies. Predicted bounding boxes and class labels illustrate each model’s ability to localize diagnostically relevant microscopic features. Numbers above bounding boxes indicate the model’s confidence for each detection.

Per-class performance analysis revealed consistent trends across all three object detection architectures. TV and GU were the most accurately detected classes (highest mAP50: 0.651–0.750), likely owing to their distinctive morphological characteristics and visual features (Figure 1 and Table 3). In contrast, HL, BV, and VVC remained more challenging to detect across all models due to their subtler diagnostic features and greater morphological heterogeneity. Specifically, HL is characterized by numerous small rod-shaped *Lactobacilli* attached to epithelial cells (Figure 2), BV by the presence of amorphous clue-cell structures, and VVC by elongated filamentous pseudohyphae (Figure 3). Despite having the largest number of annotated instances (*n* = 1,874), HL exhibited the lowest localization and classification performance, suggesting that high intra-class variability, rather than insufficient training data, was the primary factor limiting detection accuracy (Figure 2). EfficientDet-D0 performed particularly poorly for HL (Figure 2, mAP50 = 0.000) and VVC (mAP50 = 0.007), highlighting the limitations of its anchor-based architecture and lower input resolution (512 × 512 pixels versus 1,280 × 1,280 pixels) for detecting these morphologically complex and fine-grained diagnostic features within this dataset. Based on these findings, RT-DETR and YOLOv26 were selected for subsequent validation and explainability analyses.

**Table 3:** Per-class mAP50 on test dataset.

Class	Instances	YOLOv26 v2	RT-DETR v2	EfficientDet-D0
TV	89	0.750	0.748	0.275
GU	389	0.622	0.651	0.192
BV	635	0.134	0.171	0.104
HL	1,874	0.148	0.157	0.000
VVC	603	0.133	0.182	0.007

BV, bacterial vaginosis; GU, gonococcal cervicitis and/or urethritis; HL, healthy vaginal condition; TV, trichomoniasis; VVC, vulvovaginal candidiasis.

Object-level detection performance was further evaluated using precision–recall curves and average precision (AP) at four intersection-over-union (IoU) thresholds (0.1, 0.3, 0.5, and 0.7). Evaluation across multiple IoU thresholds enabled characterization of the trade-off between detection sensitivity and localization accuracy for diagnostically relevant microscopic features within each field of view. As expected, detection performance progressively declined with increasing IoU thresholds for both architectures, reflecting the greater localization precision required under more stringent matching criteria. YOLOv26 achieved a maximum recall of 0.74 at IoU = 0.1, which decreased to 0.35 at IoU = 0.7. In comparison, RT-DETR achieved a maximum recall of 0.89 at IoU = 0.1 and maintained a higher recall of 0.48 at IoU = 0.7. Similarly, the area under the precision–recall curve declined with increasing localization stringency, ranging from 0.38 to 0.10 for YOLOv26 and from 0.42 to 0.12 for RT-DETR across the evaluated IoU thresholds (Supplementary Table S2; Supplementary Figure S1). These results indicate that RT-DETR consistency outperformed YOLOv26 in both detection sensitivity and localization robustness across varying levels of spatial overlap requirements.

To assess the effect of unequal class representation, we conducted two additional RT-DETR experiments using balanced sampling and inverse-frequency class weighting (cls pw = 1.0). Compared with the original model (Supplementary Table S1, mAP50 = 0.354; mAP50–95 = 0.165), balanced sampling yielded mAP50 = 0.356 and mAP50–95 = 0.171, while class-weighted loss yielded mAP50 = 0.358 and mAP50–95 = 0.164 (Supplementary Tables S3, S4). Neither strategy substantially improved overall detection performance. Class-specific performance remained heterogeneous. The TV and GU classes showing relatively strong detection despite fewer training images, whereas BV, HL, and VVC remained more challenging.

### 2.2 Detection-based image classification

For image-level evaluation, object detection outputs were integrated into image-level classifications to reflect the intended clinical diagnostic application of the models. Using this detection-based image classification framework, both architectures achieved excellent diagnostic performance (Figure 4; Table 4). YOLOv26 achieved an overall accuracy of 95.72%, a macro F1-score of 0.9615, Cohen’s κ of 0.9433, a mean AUC of 0.9713, and a non-detection rate of 2.9%. RT-DETR achieved the accuracy of 98.25%, with a macro F1-score of 0.9807, Cohen’s κ of 0.9766, a mean AUC of 0.9846, and a substantially lower non-detection rate of 0.4%. Among the five diagnostic classes, HL demonstrated the lowest sensitivity, with values of 0.8269 (YOLO) and 0.9615 (RT-DETR), while all remaining maintained sensitivity above 0.93 and specificity above 0.99 for both models. These findings indicate robust and consistent classification performance across all diagnostic categories (Figure 4; Table 5).

**Figure 4:**
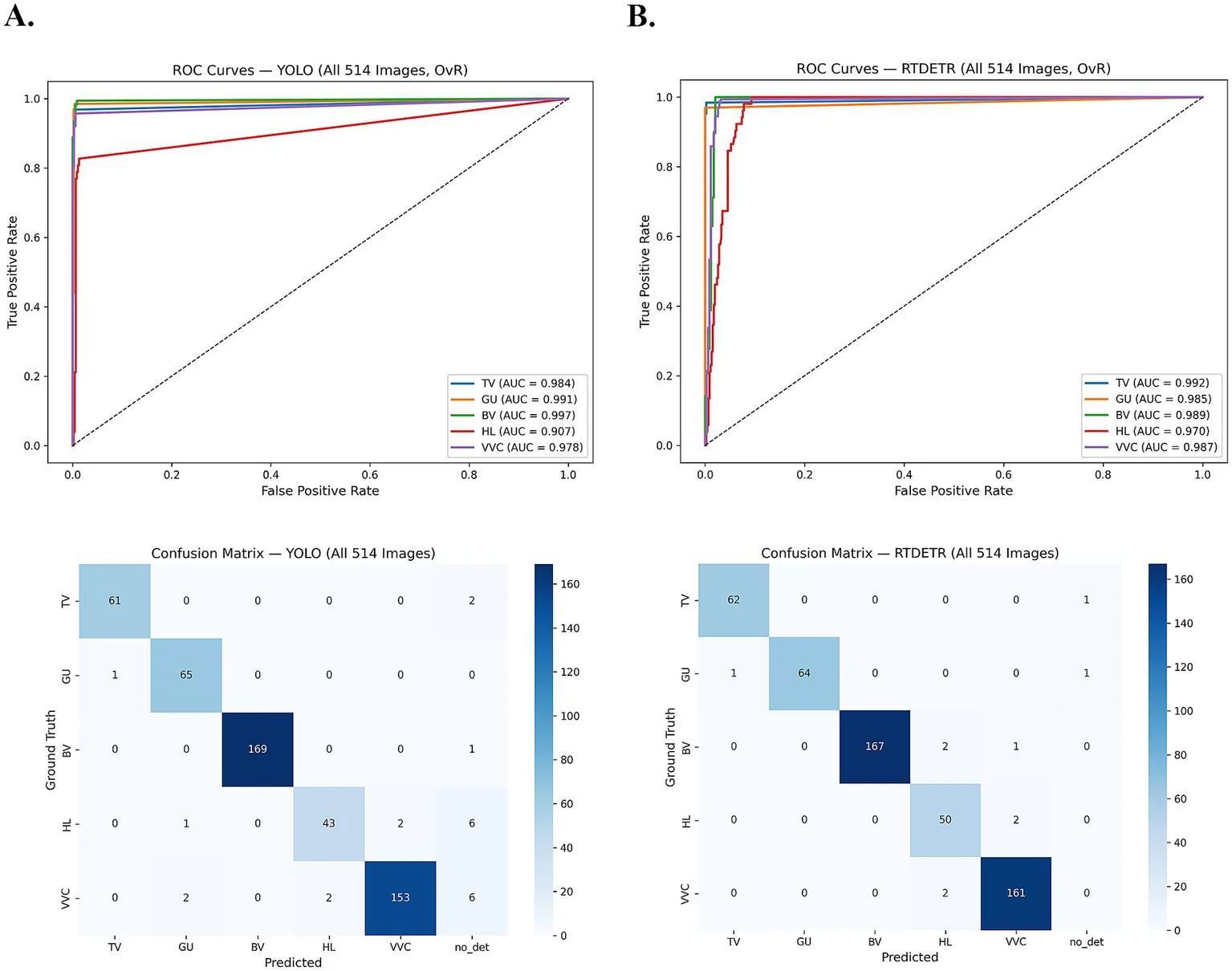
Detection-based image-level classification performance of YOLOv26 and RT-DETR on Gram-stained vaginal discharge microscopy images (*n* = 514). **(A)** Receiver operating characteristic (ROC) curves for YOLOv26 (left) and RT-DETR (right), generated using a one-vs-rest strategy across five diagnostic categories: TV, GU, BV, HL, and VVC. AUC values are reported for each class. Image-level predictions were derived using a two-step confidence-weighted any-positive rule: first, all bounding boxes exceeding a model-specific detection confidence threshold (YOLOv26 ≥ 0.1; RT-DETR ≥ 0.25) were collected; second, the diagnostic class with the highest mean confidence among detected boxes was assigned as the image-level label. ROC curves and AUC values were computed across all 514 test images, with non-detected images (YOLOv26: *n* = 15; RT-DETR: *n* = 2) assigned a confidence score of zero for all classes. **(B)** Confusion matrices for YOLOv26 (left) and RT-DETR (right) illustrate the distribution of predicted versus ground-truth image-level diagnoses across all 514 test images. Rows correspond to ground-truth classes and columns correspond to predicted classes, including a “no detection” column representing images in which no bounding box exceeded the confidence threshold. Non-detected images are scored as incorrect predictions for their respective ground-truth class. AUC, Area under the curve; BV, bacterial vaginosis; GU, gonococcal cervicitis and/or urethritis; HL, healthy vaginal condition; TV, trichomoniasis; VVC, vulvovaginal candidiasis.

**Table 4:** Performance of detection-based classification framework.

Metric	YOLO (*n* = 514)	RT-DETR (*n* = 514)
Accuracy	0.9572	0.9825
F1 Macro	0.9615	0.9807
F1 Weighted	0.9708	0.9845
Cohen’s Kappa	0.9433	0.9766
Mean AUC	0.9713	0.9846
No detection	15 (2.9%)	2 (0.4%)

All 514 images are included in this analysis; non-detection treated as incorrect. AUC, area under the receiver operating characteristic curve; F1, harmonic mean of precision and recall; N, number of images.

**Table 5:** Per-class performance for detection-based image classification.

Class	YOLO sensitivity	YOLO specificity	RT-DETR sensitivity	RT-DETR specificity	Support
TV	0.9688	1.0000	0.9844	1.0000	64
GU	1.0000	0.9933	0.9846	1.0000	65
BV	0.9941	1.0000	0.9824	1.0000	170
HL	0.8269	0.9957	0.9615	0.9913	52
VVC	0.9387	0.9943	0.9877	0.9915	163

Per-class sensitivity including non-detections (scored as false negatives). BV, bacterial vaginosis; GU, gonococcal cervicitis and/or urethritis; HL, healthy vaginal condition; TV, trichomoniasis; VVC, vulvovaginal candidiasis.

To account for multiple microscopic fields from the same specimen, RT-DETR predictions were additionally evaluated at the specimen level. The 514-image test set represented 96 unique specimens: 34 BV, 9 GU, 6 HL, 9 TV, and 38 VVC specimens (Figure 4 and Supplementary Table S5). Following image curation, 1–13 images per specimen were retained. Majority-vote aggregation produced the correct diagnosis for all 96 specimens (100%). In addition, 88/96 specimens (91.7%) had all retained microscopic fields individually classified correctly.

Error analysis identified 22 misclassified or non-detected images for YOLOv26 and 9 for RT-DETR among the 514 images in the independent test set. Three specimens were misclassified or not detected by both models, representing the most diagnostically challenging cases within the test set (Supplementary Table S6). The majority of YOLOv26 errors were attributable to non-detections (15/22), whereas RT-DETR errors were more frequently resulted from incorrect class assignment rather than failure to detect diagnostic objects. Qualitative examination of discordant cases did not identify any consistent morphological features associated with model errors (Supplementary Figure S2), suggesting that errors were not driven by a single identifiable source of visual ambiguity. Despite these challenging specimens, both models maintained high image-level diagnostic performance on the independent test set.

Notably, the image-level classification framework relies primarily on the successful detection of diagnostically relevant microscopic features rather than exhaustive localization of all annotated objects within an image. Consequently, image-level diagnostic performance is not directly dependent on conventional object detection metrics such as mAP50. The observed discrepancy between moderate object-level localization performance and excellent image-level classification accuracy supports an any-positive diagnostic paradigm, whereby the reliable detection of at least one representative diagnostic feature within a microscopic field is sufficient to establish the corresponding image-level diagnosis (Dietterich et al., 1997; Maron and Lozano-Pérez, 1997). This finding is consistent with routine microscopic practice, in which identification of key diagnostic features, rather than comprehensive enumeration of all observable structures, often determines the final clinical interpretation.

### 2.3 Explainability analysis of YOLOv26 and RT-DETR

GradCAM visualizations demonstrated spatially appropriate activation patterns for both models across representative image samples (Figure 5). In the GU specimen, model activation corresponded to regions containing localized clusters of Gram-negative diplococci within areas of dense leukocyte accumulation in the oil-immersion microscopic field (Figure 5, top). In the BV specimen, activation was concentrated around clue-cell regions, indicating appropriate localization to the principal diagnostic feature of this condition (Figure 5, bottom). RT-DETR consistently exhibited highly focal activation patterns concentrated around diagnostically relevant structures across specimen types, whereas YOLOv26 displayed broader activation regions that nevertheless remained spatially appropriate and confined to relevant areas within the microscopic field of view. These differences are consistent with the distinct feature representation and extraction mechanisms of transformer-based (RT-DETR) and convolutional (YOLOv26) architectures. Comprehensive comparisons across visualization methods and network layer are provided in Supplementary Figures S3–S5. Activation quality for YOLOv26 varied across image samples and diagnostic categories (Supplementary Figures S3, S4). Overall, both models demonstrated biologically and diagnostically plausible spatial attention patterns that aligned with relevant microscopic morphology, supporting the interpretability of their diagnostic predictions.

**Figure 5:**
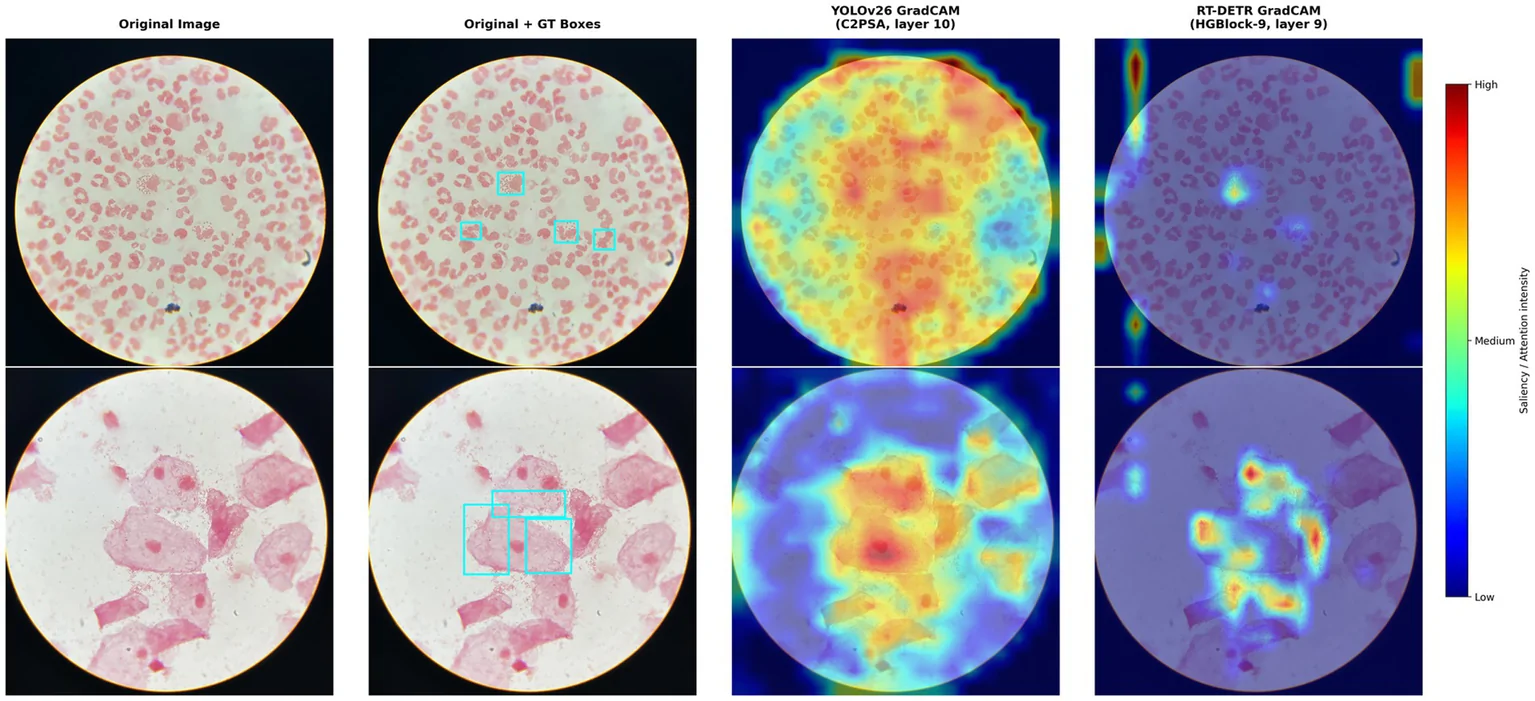
Grad-CAM visualizations for YOLOv26 and RT-DETR. Representative Gram-stained microscopic images illustrating GU (top) and BV (bottom; clue cells) are shown alongside the corresponding Grad-CAM saliency maps generated from YOLOv26 (C2PSA) and RT-DETR (HGBlock-9). In both models, the saliency maps predominantly highlighted regions corresponding to the ground-truth diagnostic features. GT, ground truth.

To quantitatively assess spatial localization, targeted Grad-CAM analysis of RT-DETR was performed on 514 test images (Figure 6 and Table 6). Overall, the mean activation enrichment was 2.40-fold (median, 1.97-fold), indicating that model activation was generally concentrated within annotated diagnostic regions relative to their image area. Enrichment varied across diagnostic categories, with the highest mean enrichment observed for GU (3.96-fold), followed by BV (3.01-fold), TV (2.35-fold), VVC (1.57-fold), and HL (1.14-fold). Pointing accuracy was 22.4% overall, reflecting the difficulty of requiring the single highest-activation pixel to fall within the annotated region, particularly for small or less abundant diagnostic structures. Notably, TV achieved the highest mAP50 but showed 0% pointing accuracy, whereas BV and VVC showed the highest pointing accuracy (38.8 and 23.9%, respectively) despite comparatively lower mAP50 values, highlighting that mAP50 alone may not fully capture the model’s spatial localization behavior. This pattern may reflect the larger size or greater abundance of diagnostic structures in these categories (clue cells and pseudohyphae), facilitating peak activation within annotated regions.

**Figure 6:**
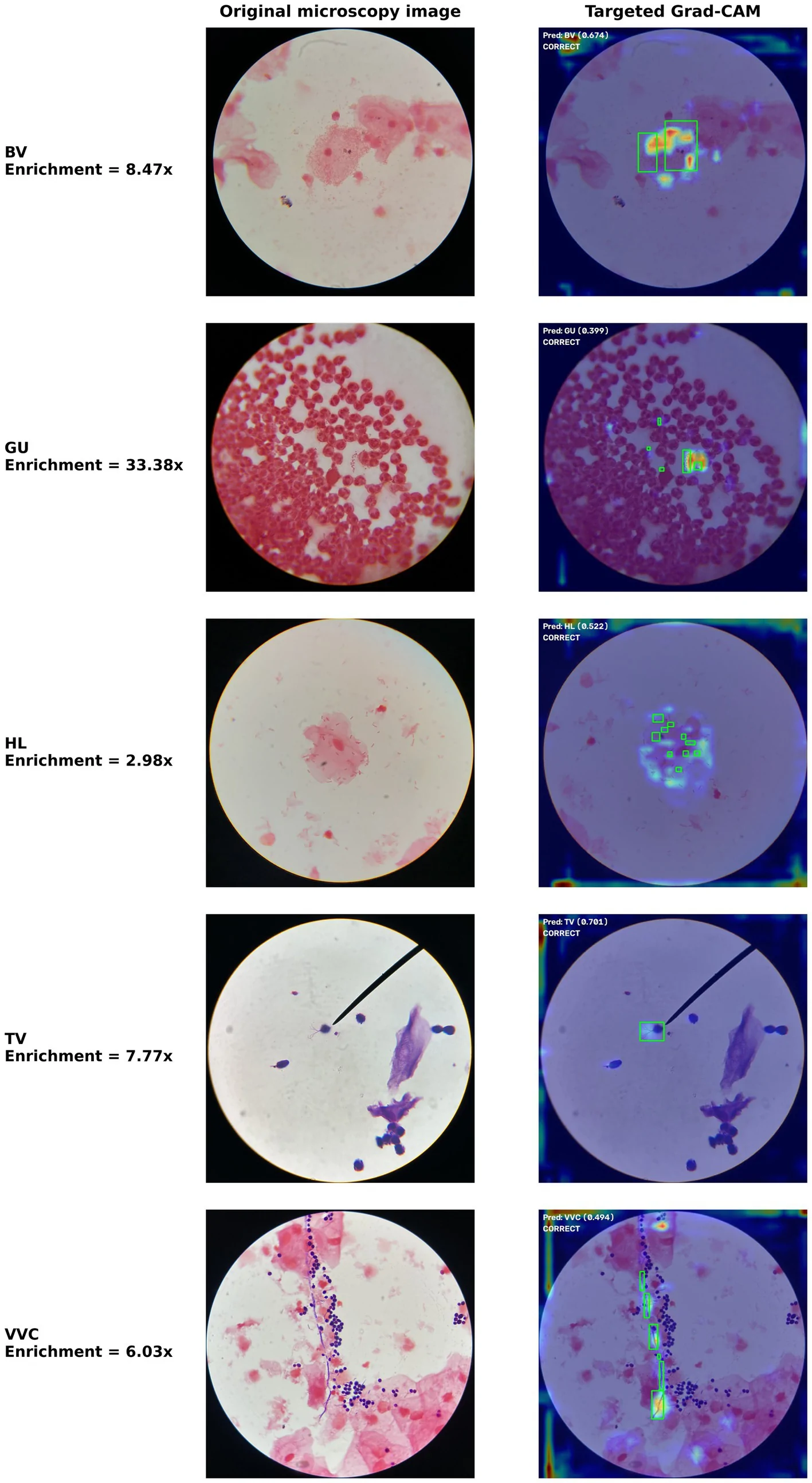
Representative Grad-CAM localization with the highest activation enrichment score for each diagnostic category. Representative RT-DETR predictions and targeted Grad-CAM visualizations for the test image with the highest activation enrichment within each diagnostic category. For each category, the original microscopy image (left) and the corresponding overlay (right) of the targeted Grad-CAM heatmap with the ground-truth bounding box are shown. Warmer colors indicate regions of higher model activation. Activation enrichment represents the proportion of CAM activation within the ground-truth diagnostic region normalized by the fraction of image area occupied by the ground-truth annotation; values >1 indicate greater-than-area-proportional activation within the annotated region. BV, bacterial vaginosis; GU, gonococcal cervicitis and/or urethritis; HL, healthy vaginal condition; TV, trichomoniasis; VVC, vulvovaginal candidiasis.

**Table 6:** Quantitative assessment of RT-DETR Grad-CAM localization on the independent test set (*n* = 514).

Diagnostic category	*n*	Mean activation inside GT	Median activation enrichment	Mean activation enrichment	Pointing accuracy
TV	64	0.0156	1.95×	2.35×	0.0%
GU	65	0.0370	2.03×	3.96×	15.4%
BV	170	0.2413	2.81×	3.01×	38.8%
HL	52	0.0203	1.16×	1.16×	0.0%
VVC	163	0.1426	1.29×	1.57×	23.9%
Overall	514	0.1337	1.97×	2.40×	22.4%

Mean activation inside GT represents the mean proportion of total Grad-CAM activation contained within the ground-truth region. Activation enrichment was calculated for each image as the activation inside GT divided by the corresponding ground-truth area fraction; median and mean enrichment were then calculated across images. Pointing accuracy represents the proportion of images in which the maximum Grad-CAM activation pixel was located within the ground-truth region. BV, bacterial vaginosis; GU, gonococcal cervicitis and/or urethritis; HL, healthy vaginal condition; TV, trichomoniasis; VVC, vulvovaginal candidiasis.

The quantitative analysis also highlighted a difficult VVC case, which was misclassified by RT-DETR and showed an activation enrichment of only 0.59-fold (Supplementary Figure S6). Visual inspection indicated that pseudohyphae, which is the diagnostic feature annotated for VVC in this study, were not clearly apparent in the image, while budding yeast forms were more evident (Supplementary Figure S6). This case illustrates how quantitative CAM localization can provide additional insight into model errors by identifying cases in which activation was poorly concentrated within the annotated diagnostic region.

### 2.4 Efficiency-accuracy benchmarking

YOLOv26 and RT-DETR models were further evaluated at five input resolutions (640, 800, 960, 1,024, and 1,280 pixels). On the held-out test set at 1280 pixels (*n* = 514 images), YOLOv26 achieved an mAP50 of 0.359 (bootstrap 95% CI: 0.340–0.379), while RT-DETR achieved an mAP50 of 0.384 (bootstrap 95% CI: 0.361–0.406). Across all input resolutions, YOLOv26 consistently exhibited lower inference latency than RT-DETR at corresponding resolutions (Figure 7A). For YOLOv26, mAP50 increased from 0.353 at 640 pixels to a peak of 0.380 at 800 pixels, followed by a slight decline at higher resolutions, reaching 0.357 at 1280 pixels. In contrast, mean inference latency increased monotonically from 28.46 ms/image at 640 pixels to 114.32 ms/image at1280 pixels (Table 7). RT-DETR showed a more consistency improvement in detection performance with increasing input resolution, with mAP50 rising from 0.327 at640 pixels to 0.381 at1280 pixels. This gain in accuracy was accompanied by a corresponding increase in mean inference latency, from 62.93 ms/image to 169.04 ms/image (Table 7).

**Figure 7:**
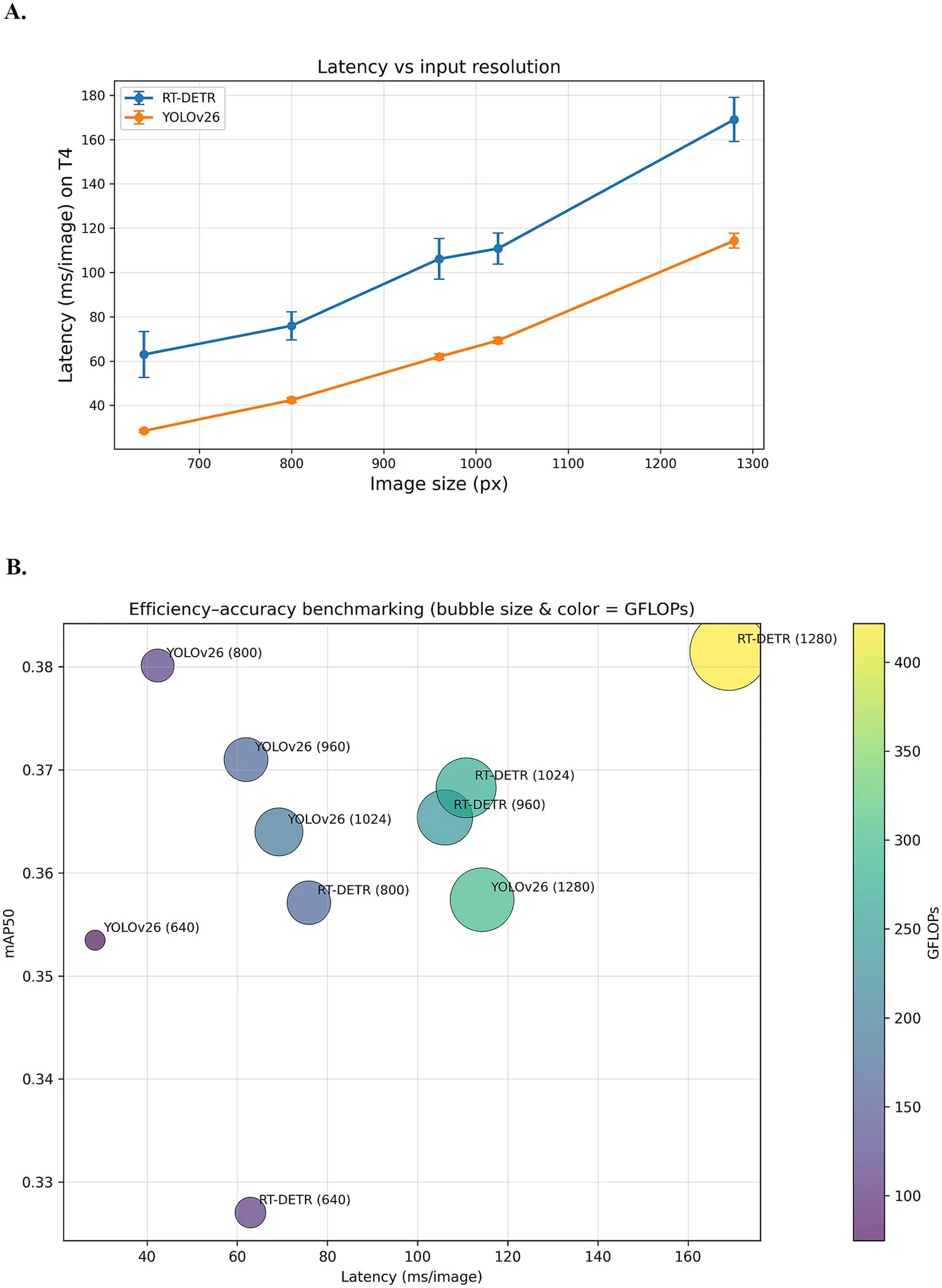
Efficiency–accuracy benchmarking across input resolutions. Model efficiency was evaluated for YOLOv26 and RT-DETR across input resolution ranging from 640 to 1,280 pixels by comparing detection accuracy (mAP50), inference speed, and computational cost. **(A)** Inference latency (ms per image) on an NVIDIA Tesla T4 GPU as a function of input resolution. Data points represent the mean latency, and error bars indicate the standard deviation across repeated measurements for each model–resolution configuration. **(B)** Accuracy–cost trade-off analysis showing mAP50 vs. GFLOPs, where GFLOPs represents the estimated number of floating-point operations required for a single forward pass at each input resolution, providing a hardware-independent measure of computational complexity. Bubble size and color are scaled according to GFLOPs to facilitate comparison of the computational demands associated with each model-resolution configuration. GFLOPs, giga floating-point operations; mAP50, mean average precision at an intersection over union threshold of 0.50; ms, milliseconds; px, pixels.

**Table 7:** Test-set detection performance (mAP50), inference latency on an NVIDIA T4 GPU (ms/image; mean ± SD), and computational complexity (GFLOPs) for YOLOv26 and RT-DETR across five input resolutions.

Model	Input resolution (pixels)	mAP50	Latency median (ms/image)	Latency mean± SD (ms/image)	GFLOPs
YOLOv26	640	0.353	28.61	28.46 ± 0.64	74.75
	800	0.380	42.34	42.34 ± 0.98	116.80
	960	0.371	61.49	61.95 ± 1.34	168.19
	1,024	0.364	68.86	69.25 ± 1.37	191.37
	1,280	0.357	113.66	114.32 ± 3.40	299.01
RT-DETR	640	0.327	56.02	62.93 ± 10.36	108.01
	800	0.357	74.08	75.90 ± 6.36	166.83
	960	0.365	100.96	106.08 ± 9.17	238.73
	1,024	0.368	108.12	110.78 ± 7.05	271.15
	1,280	0.381	163.66	169.04 ± 9.93	421.73

GFLOPs, giga floating-point operations; mAP50, mean average precision at an intersection over union threshold of 0.50; ms, milliseconds; SD, standard deviation.

Computational complexity, measured in GFLOPs, increased substantially with input resolution for both detectors, with RT-DETR consistently requiring greater computational resources than YOLOv26 across all evaluated resolutions (Table 7). At an input resolution of 640 pixels, RT-DETR required 108.01 GFLOPs compared with 74.75 GFLOPs for YOLOv26. At 1280 pixels, computational requirements increased to 421.73 GFLOPs for RT-DETR and 299.01 GFLOPs for YOLOv26. Differences in model size were also noted, with RT-DETR comprising 32.8 million parameters compared with 21.8 million parameters for YOLOv26.

### 2.5 Speed-accuracy trade-offs and selection of candidate deployment settings

Because deployment performance depends on both predictive accuracy and runtime computational efficiency, test-set mAP50 was evaluated alongside inference latency and GFLOPs (Figure 7B). RT-DETR at an input resolution of 1,280 pixels achieved the highest mAP50 (0.381) (Table 7) but also incurred the greatest computational cost (421.73 GFLOPs) and the highest mean inference latency (169.04 ms/image; median 163.66 ms/image). In contrast, YOLOv26 at 800 pixels achieved a comparable mAP50 (0.380) while requiring substantially fewer computational resources (116.80 GFLOPs) and exhibiting markedly lower inference latency (42.34 ms/image). These findings indicate that the optimal input resolution is architecture-dependent, with YOLOv26 achieving its best accuracy-efficiency trade-off at a moderate resolution, whereas RT-DETR continues to benefit from higher-resolution inputs despite the associated increase in computational cost and inference time.

### 2.6 Expert evaluation of model localization

Expert evaluation showed generally favorable localization performance for both models (Table 8). Mean localization scores were 3.35 for YOLOv26 and 3.49 for RT-DETR, with Good/Excellent ratings in 84.8 and 85.1% of valid evaluations, respectively. Inter-rater agreement was low (ordinal Krippendorff’s α = 0.134 for YOLOv26 and 0.086 for RT-DETR). In the cumulative-link mixed-effects model accounting for expert- and image-level clustering and adjusting for target class, localization ratings did not differ significantly between RT-DETR and YOLOv26 (OR for a higher rating, 0.97; 95% CI, 0.58–1.61; *p* = 0.895).

**Table 8:** Expert evaluation of model localization.

Metric	YOLOv26	RT-DETR
Mean localization score (1–4)	3.35	3.49
Good/Excellent rating (≥3)	128/151 (84.8%)	131/154 (85.1%)
Agreement within ±1 score category	82.3%	85.5%
Ordinal Krippendorff’s α	0.134	0.086
Model preference*	46.5%	53.5%
CLMM, OR for higher rating†	Reference	0.97 (95% CI, 0.58–1.61); *p* = 0.895

*Among evaluations in which either model was preferred. †Cumulative-link mixed-effects model adjusted for target class, with expert and image set as random intercepts. CI, confidence interval; CLMM, cumulative-link mixed-effects model; OR, odds ratio.

Experts generally favored bounding boxes that more completely encompassed the diagnostic structure. For example, predictions encompassing the entire pseudohypha were consistently rated as better localized in 100% of the corresponding comparisons, regardless of the model generating the prediction (Supplementary Figure S7). Overall, both models provided generally favorable localization, with no significant difference in expert-rated performance, while complete coverage of diagnostic structures was consistently associated with higher localization ratings.

## 3 Discussion

To our knowledge, this is the first report to integrate microbiota-associated conditions, sexually transmitted infections, explainable object detection, and detection-based image classification into a unified AI-assisted microscopy framework for the differential diagnosis of vaginal discharge. Unlike most previous AI approaches that focused primarily on individual or closely related pathogens, the proposed framework was designed to emulate routine microscopic interpretation by simultaneously evaluating multiple diagnostic possibilities within the same specimen while explicitly localizing diagnostically relevant microscopic structures. An additional advantage of this object detection–based framework is its inherent interpretability. Our approach explicitly localizes diagnostically relevant microscopic features, allowing clinicians to directly verify the evidence supporting each diagnosis. Explainability analyses using EigenCAM, Grad-CAM, and Grad-CAM++ further demonstrated that both models consistently focused on characteristic diagnostic structures, including pseudohyphae, clue cells, flagellated trophozoites, and intracellular Gram-negative diplococci, supporting the biological plausibility of their predictions. Compared with conventional image classification models that provide only specimen-level predictions, this explainable object detection strategy enables clinicians to visually verify the evidence supporting each diagnosis, thereby enhancing interpretability, facilitating quality assurance, and enabling systematic review of discordant or difficult cases (Ahmed et al., 2026).

A key finding of this study is the marked contrast between object-level and image-level performance. Although box-level localization performance was modest (mAP50 = 0.357 for YOLOv26 and 0.384 for RT-DETR), this likely reflects the increased complexity of simultaneously detecting five diagnostically distinct microscopic categories within a single detection framework. Nevertheless, both models achieved high image-level diagnostic performance, with an accuracy above 95%, macro F1-scores over 0.96, and mean AUC values exceeding 0.97. This disparity has important implications for evaluating detection-based AI systems in clinical microscopy. Conventional object detection metrics, such as mAP50, penalize every missed or imprecisely localized object, making them well suited for applications requiring exhaustive detection, counting, or pixel-level precise spatial localization. In contrast, routine microscopic examination of vaginal discharge is typically based on spatially recognizing diagnostic relevant microscopic features to establish the presence or absence of disease rather than detecting every annotated object within a field of view (Workowski et al., 2021; Muzny et al., 2023; Van Der Pol, 2016; Amrin and Lakshmi, 2021). Although Gram stain–based diagnosis of BV incorporates semi-quantitative assessment of bacterial morphotypes such as the Nugent score, diagnosis likewise depends on the recognition of characteristic microscopic findings, including clue cells and depletion of *Lactobacillus* morphotypes (Watanabe et al., 2025; Nugent et al., 1991). Consequently, conventional object detection metrics may underestimate the clinical utility of AI-assisted microscopy when the primary objective is specimen-level diagnosis (Varoquaux and Cheplygina, 2022; Maier-Hein et al., 2024). When the intended application is image-level diagnosis, clinically relevant performance measures, including accuracy, F1-score, AUC, and Cohen’s kappa, should be interpreted alongside object-level metrics to provide a more comprehensive assessment of diagnostic performance. This finding highlights the importance of selecting evaluation metrics that are aligned with the intended clinical application rather than relying exclusively on conventional computer vision benchmarks (Everingham et al., 2010; Lin et al., 2014, see cocodataset.org for full evaluation definitions). Overall, our study further demonstrates that clinically relevant image-level diagnosis can be achieved despite only modest object-level localization performance, supporting a presence-based rather than enumeration-based evaluation framework.

The observed differences in detection performance across diagnostic categories are biologically plausible and reflect the intrinsic morphological complexity of vaginal discharge microscopy. *T. vaginalis* trophozoites and intracellular Gram-negative diplococci possess distinctive morphological characteristics, enabling consistently high detection performance across different architectures. In contrast, bacterial vaginosis and healthy vaginal condition represent more heterogeneous biological states rather than discrete morphological entities. Bacterial vaginosis is diagnosed by recognizing complex interactions between epithelial cells and adherent bacterial communities, whereas healthy vaginal flora encompasses a broad spectrum of *Lactobacillus* abundance, epithelial cell morphology, and transitional microbiota compositions (Sadeghpour Heravi, 2024). Similarly, vulvovaginal candidiasis may present fragmented or overlapping pseudohyphae, variable staining intensity, and partial occlusion by epithelial cells or inflammatory debris (Valentine et al., 2025). These intrinsic biological and morphological variations likely contributed more to reduced detection performance than class imbalance alone, potentially explaining why healthy flora remained the most challenging category despite containing the largest number of annotated instances. This biological heterogeneity was also reflected in the error analysis. Among the 514 test images, only 22 images for YOLOv26 and 9 for RT-DETR were misclassified or non-detected, with only three specimens misclassified by both architectures. Visual inspection of these cases did not reveal a consistent morphological pattern that could account for the prediction errors. One possible contributing factor is the annotation strategy employed in this study. To facilitate image-level classification and minimize in-class label ambiguity, annotations were restricted to clear and diagnostically representative microscopic features. Consequently, faintly stained or morphologically ambiguous structures may have been underrepresented in the training data. For example, weakly stained *Candida* pseudohyphae can be difficult to distinguish from background artifacts or other filamentous structures. The limited representation of such challenging examples may have reduced model robustness when similar features were encountered in the test set, thereby contributing to the observed classification and detection errors.

Although RT-DETR achieved the highest overall object detection performance, both RT-DETR and YOLOv26 demonstrated comparable diagnostic performance on the independent test set, as reflected by their similarly high image-level classification performance. Importantly, YOLOv26 required substantially lower inference latency and computational resources than RT-DETR. These findings highlight the importance of balancing diagnostic performance with computational efficiency when considering models for smartphone-assisted microscopy, particularly in resource-limited settings where access to high-performance computing infrastructure may be constrained. Cloud-based deployment enables use of the full model, centralized updates, and browser-based access across devices but requires network connectivity. In contrast, on-device inference could enable offline operation but would require additional model optimization and device-specific evaluation, particularly on lower-end smartphones. Future studies should evaluate both deployment approaches across a range of mobile devices, considering inference latency, memory requirements, connectivity, and usability. More broadly, predictive performance should be considered alongside practical requirements for inference speed, hardware availability, and scalability when evaluating AI models for potential clinical use (Pongpom et al., 2026; Tan et al., 2020; Zhao et al., 2024).

Localization performance of each model architecture was further evaluated by eight independent experts, including clinicians and microbiologists, providing human-centered validation beyond conventional object-level metrics. Although RT-DETR achieved a modestly higher overall localization score than YOLOv26, experts showed no significant difference in localization ratings between the two architectures. Experts particularly favored bounding boxes that more completely encompassed diagnostically relevant structures. In representative comparisons, 100% of experts preferred bounding boxes that more comprehensively captured the complete diagnostic structure, illustrating that clinical localization may depend more on coverage of relevant morphology than on strict geometric conformity to annotation boundaries. Overall, these findings provide human-centered support for the clinical interpretability of the detection-based approach and its ability to provide visually meaningful diagnostic evidence for expert review.

The proposed framework has potential to support point-of-care and near-patient microscopy by combining conventional Gram-stained microscopy, smartphone-based image acquisition, and AI-assisted interpretation (Figure 8). However, the present study was designed as a retrospective proof-of-concept evaluation of smartphone-assisted microscopic feature detection and did not include prospective clinical validation. Therefore, its clinical utility and generalizability across real-world clinical settings remain to be established. This study has several limitations. First, it was conducted at a single institution using a standardized imaging workflow; therefore, formal external validation using independently collected datasets from other institutions was not performed. Differences in microscopy systems, staining procedures, laboratory protocols, smartphone devices, imaging configurations, and patient populations may affect model performance and generalizability. Future studies should evaluate the framework across multiple clinical sites, smartphone models, and imaging conditions and assess its clinical impact on diagnostic accuracy, turnaround time, and clinical decision-making. Our previous study (Pongpom et al., 2026), in which microscopic images were manually acquired by medical students using mobile devices under less controlled acquisition conditions, demonstrated robust diagnostic performance despite variability in image acquisition. Second, the available dataset did not contain documented mixed infections or overlapping diagnostic conditions; thus, the current single-label framework may not adequately represent coexisting conditions encountered in clinical practice, which should be addressed using multi-label approaches. Third, diagnostically relevant structures were annotated using bounding boxes, which may not fully capture irregular or complex morphology such as pseudohyphae and clue cells; segmentation-based annotation could provide more precise morphological delineation. Fourth, detailed clinical and demographic characteristics, including age, pregnancy status, menopausal status, symptom status, and previous antimicrobial treatment, were not available to the research team because the dataset was provided in de-identified form and could not be linked to individual clinical records. Future prospective studies should collect appropriate de-identified clinical and demographic information to enable subgroup analyses and assessment of model performance across diverse patient populations. Finally, expert evaluation was blinded and standardized but involved a limited number of experts and image sets and focused only on the highest-confidence prediction. Larger studies incorporating more experts, complete model outputs, and broader image sets will be needed to better assess localization quality and real-world clinical utility.

**Figure 8:**
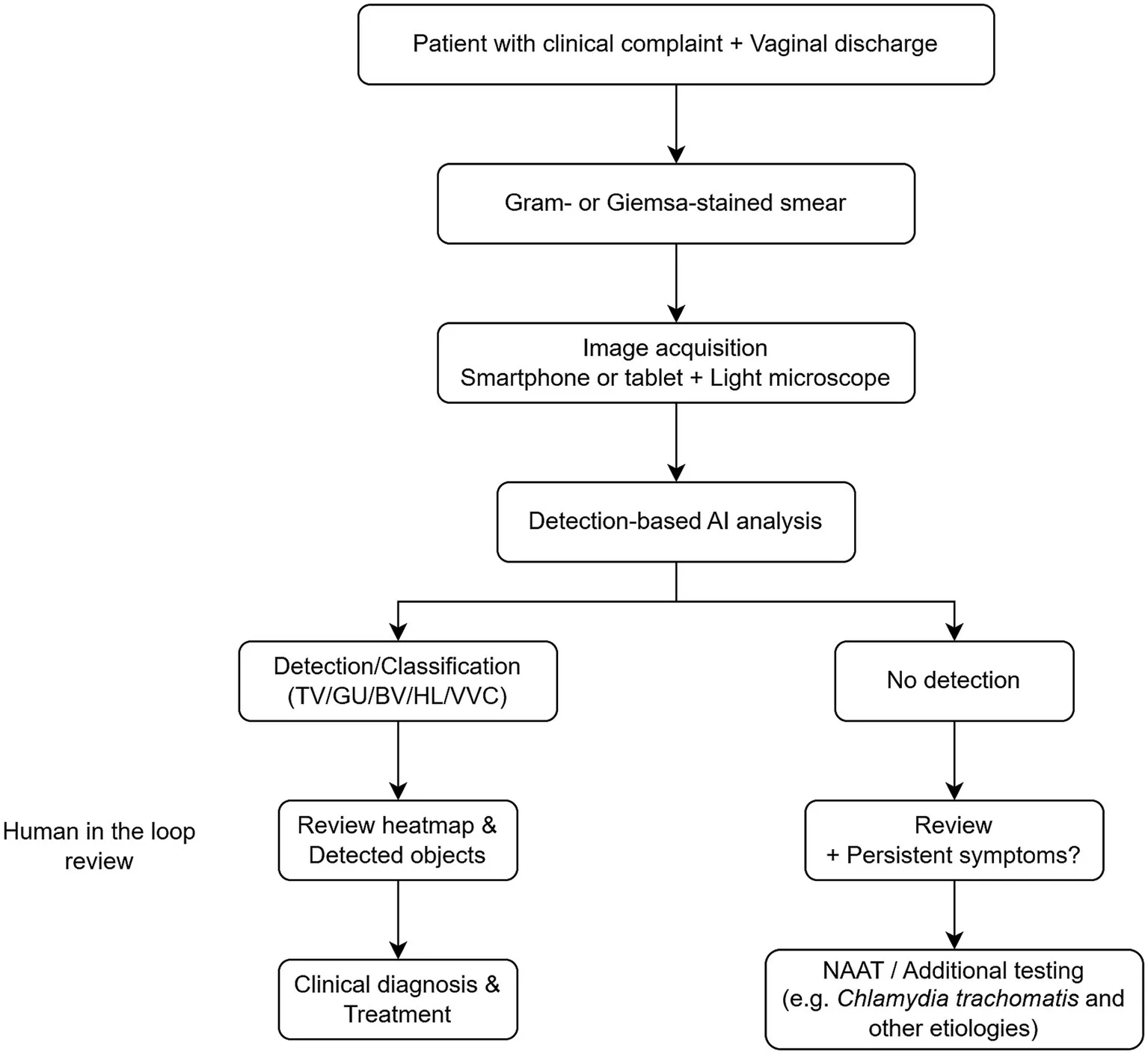
The proposed workflow illustrates the integration of AI-assisted microscopy into routine diagnostic practice as a rapid first-line decision-support tool. Patients presenting with vaginal discharge undergo standard Gram staining for BV, VVC, GU and HL-suspected samples, Giemsa staining for TV-suspected samples and microscopic examination. Images acquired using a smartphone or tablet attached to a conventional light microscope are subsequently analyzed using the proposed detection-based image classification framework. The identification of diagnostically relevant microscopic features enables image-level classification as VVC, BV, TV, GU, or HL, thereby supporting clinical interpretation and initial management decisions. In cases where no diagnostically relevant features are detected, or when clinical suspicion remains high despite negative AI-assisted microscopy findings, additional diagnostic investigations should be performed in accordance with established clinical guidelines. These may include NAATs for pathogens that are not reliably detected by Gram staining (e.g., *Chlamydia trachomatis*) or other appropriate confirmatory assays (Adamson et al., 2022). This stepwise approach allows AI-assisted microscopy to function as a rapid screening and decision-support tool while ensuring that that patients with negative, inclusive, or discordant findings receive appropriate follow-up testing and clinical evaluation. AI, artificial intelligence; BV, bacterial vaginosis; GU, gonococcal cervicitis and/or urethritis; HL, healthy vaginal condition; NAATs, nucleic acid amplification tests; TV, trichomoniasis; VVC, vulvovaginal candidiasis.

## 4 Materials and methods

### 4.1 Study design and clinical sample preparation

To evaluate performance across distinct architectural paradigms and computational requirements, three model families were selected for training and comparative analysis (Supplementary Table S7): YOLOv26, the anchor-free convolutional neural network (CNN), RT-DETR, the anchor-free vision transformer; and EfficientDet-D0, a lightweight anchor-based CNN (Tan et al., 2020; Zhao et al., 2024). This selection enabled a comprehensive assessment of diagnostic performance, computational efficiency, and deployment feasibility across a broad spectrum of architectural designs and resource constraints.

Permanent Gram-stained vaginal discharge slides were used to construct training, validation, and test datasets (Supplementary Figure S8). To ensure patient-level independence and prevent data leakage, archived microscopic slides prepared for laboratory teaching in the Human Reproductive System course for medical students were used exclusively for the training dataset. The validation and test datasets were generated from an entirely separate collection of vaginal discharge specimens, prospectively collected in September 2025–April 2026 from patients attending the Gynecology Outpatient Department, Maharaj Nakorn Chiang Mai Hospital, Chiang Mai, Thailand. The specimens were kindly provided as anonymous samples by the hospital’s Central Diagnostic Laboratory section. All patient identifiers, including names, hospital numbers, and other personal information, were removed before the specimens were provided. The research team accessed the anonymous specimens for research purposes between 26 March 2026 and 12 April 2026. The investigators did not have access to information that could identify individual participants during or after specimen collection.

The samples were subjected to Gram-staining for the diagnosis of GU, VVC, BV, and HL, whereas Giemsa staining was performed for samples with suspected TV. The HL category was defined as specimens showing no microscopic evidence of the infections evaluated in this study, with vaginal epithelial cells associated with Gram-positive bacilli consistent with Lactobacillus-dominant flora; this category was used as a reference for non-infectious vaginal findings rather than as a definitive clinical diagnosis. The specimens available for this study did not contain documented co-infections; therefore, each specimen represented a single diagnostic condition and was assigned to one of five mutually exclusive categories. Diagnostic categories were primarily established by expert microbiological assessment of the microscopic findings, with additional clinical and laboratory evidence obtained from the hospital central laboratory when the microscopic diagnosis was uncertain, including Nugent score and Amsel criteria for bacterial vaginosis, culture for vulvovaginal candidiasis and gonococcal infection, and PCR for *Trichomonas vaginalis* infection. Gram-stained microscopic images were acquired using a conventional light microscope and a Samsung Galaxy S23 Ultra smartphone. The smartphone was used without a dedicated microscope adapter and was manually aligned with the microscope eyepiece. Images were acquired using a 100X objective and 4X eyepiece, providing a total optical magnification of 400X. The same microscope configuration and illumination conditions were maintained throughout image acquisition. Five representative microscopic images, each corresponding to a different field of view, were acquired from each specimen. All slides and images were curated and independently interpreted by a clinical microbiology expert in vaginal discharge microscopy (S. S.). No specimen or patient contributed images to more than one dataset (training, validation, or test), thereby ensuring complete patient-level separation between datasets.

### 4.2 Image annotation

All microscopic images were annotated and independently reviewed by at least two annotators according to established diagnostic criteria. Annotations were generated using the Labelbox platform (Labelbox, Inc.) by delineating diagnostically relevant features with bounding boxes for TV, GU, BV, and HL and with free form segmentation for VVC. Subsequently, the expert (S. S.) reviewed all annotations and adjudicated any discrepancies to establish a consensus ground truth. Formal inter-observer agreement statistics were not calculated. The finalized annotations were exported from Labelbox in JSON format and converted into the required YOLO training formats using custom Python scripts as previously described (Pongpom et al., 2026).

### 4.3 Annotation format

Images and their corresponding object annotations were organized using a YOLO-based directory structure comprising training, validation, and test subsets. Ground-truth annotations were stored in YOLO text format (.txt), with a single annotation file associated with each image. Each annotated object was represented by a class index and normalized bounding box coordinates, including the center x-coordinate, center y-coordinate, width, and height. Dataset paths and class definitions were specified in a configuration file (data.yaml), which included the dataset root directory (path), image directories for each subset (train, validation, test), the number of classes (nc = 5), and the corresponding class names (names).

### 4.4 Dataset partitioning and model training

The dataset comprised 1,966 microscopic images containing 11,069 annotated instances across five diagnostic categories (Table 1). To prevent data leakage and ensure patient-level independence, images were partitioned at patient levels into training (*n* = 1,003), validation (*n* = 449), and test (*n* = 514) sets. The training set was used for model fitting, the validation set for hyperparameter optimization and model selection, and the independent test set for final performance evaluation. The training, validation, and test sets contained 4,174, 3,590, and 3,305 annotated instances, respectively (Table 1). YOLOv26 and RT-DETR were trained using the Ultralytics framework with an input image size of 1,280 × 1,280 pixels. To improve robustness to variation in microscopic image appearance, training images were augmented using the Ultralytics pipeline with random rotation (±10°), scaling (0.5), horizontal and vertical flipping (probabilities 0.5 and 0.1), and HSV-based photometric variation (hue = 0.015, saturation = 0.7, brightness = 0.4). Mosaic augmentation was applied during training and disabled for the final 20 epochs; MixUp, explicit random cropping, and noise injection were not used. Representative augmented images were visually inspected to ensure realistic appearances and preservation of diagnostically relevant morphology. EfficientDet-D0 was trained using a separate implementation with an input image size of 512 × 512 pixels. Hyperparameter were optimized independently for each architecture using the validation set, and the model weights corresponding to the best validation performance were selected for evaluation on the independent test set. These resulting optimized models were subsequently used for explainability analysis, inference-latency benchmarking and computational complexity assessment.

The primary models were trained using the natural class distribution without class-weighted loss or oversampling/balanced sampling. To assess the potential effect of class imbalance, two additional RT-DETR models were trained using inverse-frequency image-level balanced sampling with replacement and class-weighted classification loss (cls_pw = 1.0). The validation and test sets were kept unchanged, and performance was compared using precision, recall, and mAP50.

### 4.5 Evaluation and performance metrics

Model performance was evaluated on the held-out test set comprising 514 images using mAP at IoU threshold of 0.5 (mAP50). mAP50 was selected as the primary evaluation metric because it can be computed consistently across all three model architectures, enabling direct comparison of object detection performance. Because EfficientDet-D0 was evaluated using an inference pipeline different from that used for the Ultralytics-based models, precision and recall metrics were not directly comparable across architectures and were therefore excluded from cross-model comparisons.

To quantify uncertainty in model performance estimates, a non-parametric bootstrap procedure was performed at the image level. For each model, predictions were generated once for all test images and subsequently stored. Bootstrap resampling of test set was then conducted with replacement for 1,000 replicates (*B* = 1,000). For each replicate, mAP50 was recomputed on the resampled image set using COCO-style evaluation. The 95% confidence interval (CI) was estimated using the percentile method and defined by the 2.5th and 97.5th percentiles of the resulting bootstrap distribution.

### 4.6 Box-level precision-recall analysis

To assess bounding box detection performance, predicted bounding boxes were matched to ground-truth annotations using IoU metric. A true positive was defined as a predicted bounding box assigned to the correct class and achieving an IoU greater than a specified threshold with a corresponding ground-truth annotation. Precision-recall curves were generated by varying the confidence threshold from 0.01 to 0.99 and evaluated at four IoU thresholds (0.1, 0.3, 0.5, 0.7) (Koo et al., 2021; Ren et al., 2025).

### 4.7 Detection-based image classification

Object detection models inherently generate bounding box predictions rather than image-level diagnoses. To derive image-level classification from detection outputs, a two-step confidence-weighted any-positive rule was applied. In the first step, all predicted bounding boxes exceeding a model-specific detection confidence threshold (YOLOv26: conf ≥ 0.1; RT-DETR: conf ≥ 0.25) were collected; any diagnostic class with at least one box above this threshold was considered detected within the image. In the second step, the mean confidence score was computed across all detected boxes for each class, and the image was assigned the diagnostic class with the highest mean confidence. This approach is conceptually related to the standard assumption in Multiple Instance Learning (MIL), in which the presence of a single positive instance is sufficient to assign a positive label to the entire image (Dietterich et al., 1997; Maron and Lozano-Pérez, 1997), and similar instance-to-image aggregation strategies have been applied in computational pathology (Campanella et al., 2019). Unlike weakly supervised MIL methods that learn only from image-level labels (Campanella et al., 2019), our models were trained using fully supervised bounding box annotations, with image-level diagnoses derived from instance-level detections only during inference.

Confidence thresholds were selected via a sweep from 0.05 to 0.50 on the test set (Supplementary Table S8; Supplementary Figure S9). For YOLOv26, performance was stable from conf = 0.05–0.10 (F1 macro = 0.9807, coverage = 97.1%), with conf = 0.10 retained as the operational threshold. For RT-DETR, performance was stable from conf = 0.05–0.25 (F1 macro = 0.9838, coverage = 99.6%), with conf = 0.25 selected accordingly. Thresholds were chosen to maximize image coverage while maintaining classification accuracy, consistent with the clinical priority of minimizing missed diagnoses. Images with no detections above threshold were recorded as indeterminate and are recommended for manual microscopic review in clinical deployment.

Neither the number nor the size of detected bounding boxes was incorporated into the classification decision. Model performance was assessed at the image level using accuracy, macro-averaged and weighted F1-score, Cohen’s Kappa coefficient, and class-specific sensitivity and specificity. Receiver operating characteristic (ROC) curves were generated using a one-vs-rest (OvR) strategy, whereby each class was evaluated independently against all remaining classes. The area under the ROC curve (AUC) was calculated for each class using the mean detection confidence across all bounding boxes assigned to that class as the ranking score. The mean AUC across all five classes was subsequently reported. All analyses were conducted in Python using the Ultralytics, scikit-learn, and seaborn libraries. For error analysis, misclassified and non-detected images from each model were extracted and subjected to visual review. Predicted bounding boxes were overlaid on the original images together with corresponding activation heatmaps to facilitate qualitative assessment of model performance and provide insights into potential failure modes.

### 4.8 Specimen-level evaluation

To account for the non-independence of multiple microscopic fields obtained from the same specimen, image-level predictions, obtained from detection-based image classification method, were additionally aggregated at the specimen level. Predictions were grouped according to specimen identifier, and the specimen-level diagnosis was determined by majority vote across all retained images from each specimen. Specimen-level accuracy was calculated as the proportion of specimens for which the aggregated prediction agreed with the reference diagnosis. As a measure of within-specimen prediction consistency, we additionally calculated the proportion of specimens for which all retained microscopic fields were individually classified correctly.

### 4.9 Explainability analysis

To assess the spatial reasoning and feature localization behavior, Explainable AI (XAI) visualizations were generated using the *pytorch-grad-cam* library (v0.4.x). Several gradient- and activation-based XAI methods, including EigenCAM, GradCAM, and GradCAM++ (Muhammad and Yeasin, 2020; Selvaraju et al., 2017; Chattopadhay et al., 2018), were systematically evaluated across multiple architectural stages within each model, including backbone, post-attention, and neck modules. For qualitative visualization, the C2PSA module (layer 10) was selected for YOLOv26, whereas the HGBlock-9 (layer 9) was selected for RT-DETR. These layers represent deep feature representations and produced visually interpretable activation patterns suitable for assessing correspondence with diagnostically relevant microscopic structures. Input images were resized to 640 × 640 pixels, consistent with the inference configuration used during model evaluation. Activation heatmaps were generated and superimposed onto the original images using a *jet* colormap, where warmer colors indicate regions contributing more strongly to model prediction and cooler colors indicate areas of lower relevance. The resulting visualizations were used to qualitative assess whether model attention was focused on diagnostically meaningful structures and to compare localization behavior across architectures.

To quantitatively assess spatial localization, targeted Grad-CAM was applied to RT-DETR on the independent test set using the RepC3 module (layer 24). For each image, the target was defined by the predicted diagnostic class and highest-scoring corresponding decoder query. CAM activation was compared with ground-truth (GT) bounding boxes using activation inside GT, activation enrichment (activation inside GT normalized by the GT area fraction; >1 indicates preferential activation within GT regions), and pointing accuracy (maximum CAM activation located within a GT region). These metrics were interpreted as measures of localization agreement rather than explanation faithfulness.

### 4.10 Efficiency–accuracy benchmarking

Inference efficiency was evaluated on an NVIDIA Tesla T4 GPU using CUDA acceleration. End-to-end prediction latency was measured on a per-image basis using Ultralytics inference API (*model.predict*). To reduce variability associated with data loading and storage access, 50 test images were preloaded into memory prior to benchmarking. For each model–resolution configuration, inference was preceded by 30 warm-up iterations that were excluded from timing measurements, followed by 100 timed iterations. GPU execution was synchronized using *torch.cuda.synchronize* immediately before and after each inference call to ensure accurate latency measurement. Latency was reported as the mean ± standard deviation across timed iterations, with the median latency also recorded to characterize central tendency and robustness to outliers. Computational complexity was estimated in the terms of Giga Floating-Point Operations (GFLOPs) using the THOP (*pytorch-OpCounter*) profile framework. Profiling was performed on a single dummy input tensor with dimensions 1 × 3 × *imgsz* × *imgsz* for each model and resolution configuration. FLOPs were calculated according to the standard convention of twice the number of multiply-accumulate operation (FLOPs = 2 × MACs). Model size was quantified by the total number of trainable parameters obtained directly from the PyTorch model definition, while THOP-derived parameter counts were recorded as a consistency check.

### 4.11 Expert evaluation

An expert evaluation was conducted to assess the clinical appropriateness of bounding-box localization produced by two object-detection models (YOLOv26 and RT-DETR) on microscopic images of vaginal discharge. Twenty image sets were evaluated (four per target finding: trophozoites, clue cells, epithelial-attached lactobacilli, intracellular Gram-negative diplococci, and pseudohyphae) using a web-based survey (Google Forms). Expert evaluation participants were prospectively recruited from 24 July 2026 to 24 August 2026. Participants provided informed consent electronically before accessing the evaluation questionnaire. Eight experts (six clinicians and two microbiologists) completed the evaluation; four reported more than 5 years of microscopy and diagnostic experience, two reported 1–3 years, and two reported less than 1 year of experience. All participants were adults. The study protocol was reviewed and approved by the institutional ethics committee (Approval No. 441/2568).

For each image set, predictions from YOLOv26 and RT-DETR were displayed using anonymized labels (Model A and Model B), with model-to-label assignment randomized across image sets to blind participants to model identity. To standardize comparisons and avoid confounding from differences in detection box counts between models, only the single highest-confidence bounding box for the target class was displayed for each model. Experts rated localization appropriateness using a 4-point ordinal scale (Poor, Fair, Good, and Excellent), with an additional option for “No target present/Not applicable,” and selected which model showed better localization. Ratings of “No target present/Not applicable” were summarized descriptively and excluded from the ordinal localization-score analysis.

Inter-rater agreement was assessed separately for each model using ordinal Krippendorff’s alpha. Localization ratings were compared using a cumulative-link mixed-effects model, with model type and target class as fixed effects and expert and image set as random intercepts. Model effects were reported as odds ratios (ORs) with 95% confidence intervals.

### 4.12 Hardware and software

All model training experiments were conducted on the CMU ERAWAN High-Performance Computing (HPC) cluster using an NVIDIA A100-SXM4-80GB GPU. Data visualization, inference latency benchmarking and computational complexity analyses were conducted in a cloud-based Python environment equipped with an NVIDIA Tesla T4 GPU. Model training, validation, and inference were implemented using the Ultralytics deep learning framework. Data processing, statistical analysis and figure generation were conducted using standard scientific Python libraries, including NumPy, pandas, and Matplotlib.

## 5 Conclusion

We developed an explainable deep learning framework for simultaneous microscopic detection of vulvovaginal candidiasis, bacterial vaginosis, trichomoniasis, gonococcal infection, and healthy vaginal flora using smartphone-acquired images of Gram-stained vaginal smears captured through a conventional light microscope. The detection-based image classification strategy achieved high image-level diagnostic performance while providing explicit localization of diagnostically relevant microscopic features. Although RT-DETR showed slightly superior object-level detection performance, both architectures demonstrated comparable image-level diagnostic performance, while YOLOv26 showed a favorable balance of diagnostic performance, inference speed, and computational efficiency that may be advantageous for resource-constrained applications. Explainability analyses and independent expert evaluation further supported the interpretability and visual relevance of AI-generated localization. Collectively, these findings demonstrate the potential of smartphone-assisted AI microscopy as an interpretable clinical decision-support prototype for vaginal discharge diagnosis. By leveraging conventional microscopy infrastructure and mobile devices, this framework provides a foundation for accessible AI-assisted microscopy. However, prospective multicenter studies with rigorous specimen-level external validation across varied imaging equipment, staining protocols, operators, and clinical settings are required before broader clinical implementation or autonomous diagnostic use can be considered.

## Data Availability

The datasets presented in this article are not readily available because of ethical and institutional restrictions on the sharing of clinical specimen-derived microscopic images. Although patient-identifying information was removed from the dataset, approval for unrestricted public dissemination of the images was not obtained. The source code for the detection-based image classification and quantitative Grad-CAM localization analyses is publicly available at GitHub repository (https://github.com/N-Lawan/Detection-based-image-classification-microscopy).
