# Supplementary material for: Interpretable detection-based image classification for differential diagnosis of vaginal discharge using smartphone-assisted microscopy
Source: Front Artif Intell. 2026 Oct 15;9:1985517. doi: 10.3389/frai.2026.1985517 (PMC13716771; doi:10.3389/frai.2026.1985517)
Supplement: Supplementary file 1 [file Data_Sheet_1.docx]

Supplementary Material

Interpretable Detection-Based Image Classification for Differential Diagnosis of Vaginal Discharge Using Smartphone-Assisted Microscopy

**Siriwoot Sookkhee^1^, Marut Wongtapin^1^, Trin Phuriyaphan^1^, Kornkaran Tonpiriyaphong^1^, Nitan Wachirapaet^1^, Surapee Wasanapitanont^1^, Jidapa Joysa^1^, Monsicha Pongpom^1^, Narin Lawan^2^, Phit Upaphong^3*^, Tanaporn Wangsanut^1*^**

^1^Department of Microbiology, Faculty of Medicine, Chiang Mai University, Chiang Mai, Thailand

^2^Department of Chemistry, Faculty of Science, Chiang Mai University, Chiang Mai, Thailand

^3^Department of Ophthalmology, Faculty of Medicine, Chiang Mai University, Chiang Mai, Thailand

*** Correspondence:**
Corresponding Author
phit.u@cmu.ac.th, twangsanut@gmail.com

**Table S1.** Evaluation results on validation dataset.

| **Metric** | **YOLOv26 v2** | **RT-DETR v2** | **EfficientDet-D0** |
| --- | --- | --- | --- |
| **Precision** | 0.38 | 0.368 | - |
| **Recall** | 0.386 | 0.437 | - |
| **mAP50** | 0.339 | 0.354 | 0.1 |
| **mAP50-95** | 0.157 | 0.165 | 0.028 |

**Abbreviations:** mAP50, mean average precision at an intersection over union (IoU) threshold of 0.50; mAP50–95, mean average precision averaged across IoU thresholds from 0.50 to 0.95.

**Table S2.** Precision-recall analysis across different IoU.

| **Model** | **IoU** | **AUC (≈AP)** | **Max Recall** |
| --- | --- | --- | --- |
| RT-DETR | 0.1 | 0.423 | 0.891 |
|  | 0.3 | 0.323 | 0.820 |
|  | 0.5 | 0.228 | 0.710 |
|  | 0.7 | 0.117 | 0.479 |
| YOLO | 0.1 | 0.378 | 0.743 |
|  | 0.3 | 0.276 | 0.650 |
|  | 0.5 | 0.197 | 0.542 |
|  | 0.7 | 0.102 | 0.350 |

**Abbreviations:** AP, average precision; AUC, area under the precision–recall curve; IoU, intersection over union.

**Figure S1. Box-level precision-recall curves for YOLOv26 (left) and RT-DETR (right) on Gram-stained vaginal discharge microscopy images (n=514; 3,305 annotated instances).** Precision-recall curves were constructed by varying the detection confidence threshold from 0.01 to 0.99 at four IoU thresholds (0.1, 0.3, 0.5, and 0.7). A true-positive was defined as a predicted bounding box assigned to the correct diagnostic class and achieving an IoU ≥ threshold to the specified threshold with an unmatched ground-truth annotation. Matching was performed greedily in descending order of prediction confidence. Curves represent macro-averaged precision and recall across all five diagnostic categories. The area under the precision-recall curve (AUC) for each IoU threshold is reported in Table S3. **Abbreviation:** IoU, intersection over union.

**Table S3. Overall performance of three RT-DETR experiments addressing class imbalance.** Evaluation results on the validation dataset.

| **RT-DETR training strategy** | **Precision** | **Recall** | **mAP50** | **mAP50-95** |
| --- | --- | --- | --- | --- |
| Original | 0.368 | 0.437 | 0.354 | 0.165 |
| Balanced sampling | 0.385 | 0.461 | 0.356 | 0.171 |
| Class-weighted loss (cls pw=1.0) | 0.394 | 0.440 | 0.358 | 0.164 |

**Abbreviations:** RT-DETR, Real-Time Detection Transformer; mAP, mean average precision; mAP50, mean average precision at an intersection-over-union (IoU) threshold of 0.50; mAP50–95, mean average precision averaged across IoU thresholds from 0.50 to 0.95; cls pw, classification positive weight.

**Table S4. Per-class performance of three RT-DETR experiments addressing class imbalance.** Evaluation results on the validation dataset.

| **Class** | **Original mAP50** | **Balanced sampling** | **Class-weighted** |
| --- | --- | --- | --- |
| TV | 0.891 | 0.910 | 0.907 |
| GU | 0.382 | 0.406 | 0.403 |
| BV | 0.146 | 0.128 | 0.145 |
| HL | 0.193 | 0.184 | 0.188 |
| VVC | 0.157 | 0.152 | 0.149 |

**Abbreviations:** BV, bacterial vaginosis; GU, gonococcal cervicitis and/or urethritis; HL, healthy vaginal condition; TV, trichomoniasis; VVC, vulvovaginal candidiasis.

**Table S5**. Specimen-level evaluation of RT-DETR on the test dataset (n = 514).

| **Diagnostic category** | **Specimens** | **Majority-vote correct** | **Specimen accuracy** | **All fields correct** |
| --- | --- | --- | --- | --- |
| BV | 34 | 34 | 100% | 31/34 (91.2%) |
| GU | 9 | 9 | 100% | 8/9 (88.9%) |
| HL | 6 | 6 | 100% | 5/6 (83.3%) |
| TV | 9 | 9 | 100% | 8/9 (88.9%) |
| VVC | 38 | 38 | 100% | 36/38 (94.7%) |
| **Overall** | **96** | **96** | **100%** | **88/96 (91.7%)** |

**Abbreviations:** BV, bacterial vaginosis; GU, gonorrhea; HL, healthy vaginal condition; TV, trichomoniasis; VVC, vulvovaginal candidiasis.

**Table S6.** Images that are misclassified or not detected by both models

| **Image** | **GT** | **YOLO** | **RT-DETR** | **Pattern** |
| --- | --- | --- | --- | --- |
| B27 (P3 HL)_A.jpg | HL | BV | VVC | Both wrong, different predictions |
| S039 (TV) (3)_A.jpg | TV | no-detection | no-detection | Both missed completely |
| V030 (VVC) (1)_B.jpg | VVC | HL | HL | Both agree on wrong class |

**Abbreviations:** BV, bacterial vaginosis; GT, ground truth; GU, gonococcal cervicitis and/or urethritis; HL, healthy vaginal condition; TV, trichomoniasis; VVC, vulvovaginal candidiasis.

**Table S7.** Architecture summary.

| **Property** | **YOLOv26** | **RT-DETR** | **EfficientDet-D0** |
| --- | --- | --- | --- |
| **Architecture** | Anchor-free CNN | Anchor-free Transformer | Anchor-based CNN |
| **Parameters** | 20.35M | 31.99M | 3.83M |
| **GFLOPs** | 67.9 | 103.5 | 2.5 |
| **Input resolution** | 1280 x 1280 pixels | 1280 x 1280 pixels | 512 x 512 pixels |
| **Anchor type** | Anchor-free | Anchor-free | Anchor-based |

**Abbreviations:** CNN, convolutional neural network; GFLOPs, giga floating-point operations; M, million; px, pixels.

**Table S8.** Confidence threshold sweep for YOLOv26 and RT-DETR across the test set (n=514).

| **Model** | **Conf** | **F1 (detected only)** | **Acc (detected only)** | **F1 (all 514)** | **Acc (all 514)** | **Coverage** | **No-det** |
| --- | --- | --- | --- | --- | --- | --- | --- |
| **YOLO** | 0.05 | 0.9807 | 0.9860 | 0.9615 | 0.9572 | 97.1% | 15 |
|  | **0.10** | **0.9807** | **0.9860** | **0.9615** | **0.9572** | **97.1%** | **15** |
|  | 0.15 | 0.9880 | 0.9910 | 0.9106 | 0.8580 | 86.6% | 69 |
|  | 0.20 | 0.7948 | 0.9934 | 0.6651 | 0.5856 | 58.9% | 211 |
|  | 0.25 | 0.7928 | 0.9900 | 0.5338 | 0.3872 | 39.1% | 313 |
|  | 0.30 | 0.7941 | 0.9932 | 0.4437 | 0.2840 | 28.6% | 367 |
|  | 0.35 | 0.7918 | 0.9916 | 0.3870 | 0.2296 | 23.2% | 395 |
|  | 0.40 | 0.7848 | 0.9894 | 0.3312 | 0.1809 | 18.3% | 420 |
|  | 0.50 | 0.5308 | 0.9821 | 0.2270 | 0.1070 | 10.9% | 458 |
| **RTDETR** | 0.05 | 0.9838 | 0.9863 | 0.9807 | 0.9825 | 99.6% | 2 |
|  | 0.10 | 0.9838 | 0.9863 | 0.9807 | 0.9825 | 99.6% | 2 |
|  | 0.15 | 0.9838 | 0.9863 | 0.9807 | 0.9825 | 99.6% | 2 |
|  | 0.20 | 0.9838 | 0.9863 | 0.9807 | 0.9825 | 99.6% | 2 |
|  | **0.25** | **0.9838** | **0.9863** | **0.9807** | **0.9825** | **99.6%** | **2** |
|  | 0.30 | 0.9852 | 0.9881 | 0.9708 | 0.9689 | 98.1% | 10 |
|  | 0.35 | 0.9766 | 0.9883 | 0.8608 | 0.8210 | 83.1% | 87 |
|  | 0.40 | 0.9291 | 0.9902 | 0.6779 | 0.5875 | 59.3% | 209 |
|  | 0.50 | 0.8000 | 1.0000 | 0.4279 | 0.2665 | 26.7% | 377 |

Bold = selected threshold. F1 and Accuracy reported for both detected-only and all 514 images.

**Abbreviations:** Conf, confidence threshold; F1, F1 score; Acc, accuracy; Coverage, proportion of images with a detected prediction; No-det, images with no detection.

**Figure S2. Representative misclassified and non-detected specimens identified during error analysis.** Images illustrate diagnostically challenging cases that resulted in incorrect image-level classification or failure of detection by YOLOv26 and/or RT-DETR. Qualitative visual inspection did not reveal any consistent morphological characteristics associated with model prediction errors, suggesting that misclassifications were not attributable to a single identifiable source of visual ambiguity. Numbers above bounding boxes indicate the model's confidence for each detection. The Grad-CAM heat map predominantly focused on pseudohyphae, the characteristic morphological feature used to diagnose vulvovaginal candidiasis (VVC), consistent with the ground truth. **Abbreviations:** Conf, confidence; GT, ground truth; GU, gonococcal cervicitis and/or urethritis; Pred, prediction; VVC, vulvovaginal candidiasis.

**Figure S3. Visualization of YOLOv26 explainability outputs across layers and explainability methods.** A representative Gram-stained microscopic image of VVC is shown with heatmaps generated using EigenCAM, Grad-CAM, and Grad-CAM++. For each explainability method, activation maps are presented for multiple YOLOv26 feature layers, including C3k2-8 (the final convolutional layer of the backbone), SPPF-9 (spatial pyramid pooling features), C2PSA-10 (features processed by the PSA attention module), Neck-16 (higher-resolution neck features), and Neck-22 (lower-resolution, semantically enriched neck features). The heatmaps illustrate the spatial distribution of model attention across different network layers and explainability methods, highlighting the image regions that contribute most strongly to the model’s predictions. The GradCAM and GradCAM++ heatmaps from the C3k2-8, SPPF-9, and C2PSA-10 layers predominantly highlighted pseudohyphae and budding yeast, the characteristic morphological features of VVC, whereas the remaining heatmaps exhibited more diffuse or nonspecific activation. **Abbreviations:** VVC, vulvovaginal candidiasis; XAI, explainable artificial intelligence.

**Figure S4. Visualization of YOLOv26 explainability outputs across layers and explainability methods.** A representative Gram-stained microscopic image of TV is shown with heatmaps generated using EigenCAM, Grad-CAM, and Grad-CAM++. For each explainability method, activation maps are presented for multiple YOLOv26 feature layers, including C3k2-8 (the final convolutional layer of the backbone), SPPF-9 (spatial pyramid pooling features), C2PSA-10 (features processed by the PSA attention module), Neck-16 (higher-resolution neck features), and Neck-22 (lower-resolution, semantically enriched neck features). The heatmaps illustrate the spatial distribution of model attention across different network layers and explainability methods, highlighting image regions that contribute most strongly to the model’s predictions. In this example, the EigenCAM at C3k2-8, SPPF-9 and C2PSA-10 layers and GradCAM++ at C3k2-8 heatmaps demonstrated a localization trend toward *Trichomonas vaginalis* and cellular debris across. **Abbreviations:** TV, trichomoniasis; XAI, explainable artificial intelligence.

**Figure S5. Visualization of RT-DETR explainability outputs across layers and explainability methods.** A representative Gram-stained microscopic image of TV is shown with heatmap generated using EigenCAM, Grad-CAM, and Grad-CAM++. For each explainability method, maps are shown for multiple RT-DETR feature layers, including HGBlock-9 (backbone convolution features before preceding the transformer encoder), Conv-10 (compressed features input to the AIFI module), RepC3-21 (fine-scale, higher-resolution neck features), and RepC3-27 (coarse-scale, semantically enriched neck features). The heatmaps illustrate the spatial distribution of model attention across different network layers and explainability methods, highlighting image regions that contribute most strongly to the model’s predictions. In this example, only GramCAM at HGBlock-9 layer correctly localize to the *Trichomonas vaginalis*. **Abbreviations:** TV, trichomoniasis; XAI, explainable artificial intelligence.

**Figure S6. Explainability analysis of a misclassified VVC case by RT-DETR.** A representative error case, V030 (VVC) (1)_B.jpg, showing (A) the original microscopy image with the ground-truth VVC bounding box, (B) the original image with the RT-DETR prediction of healthy vaginal condition (HL), (C) the targeted Grad-CAM heatmap, and (D) the Grad-CAM overlay with the ground-truth bounding box. The activation enrichment for the VVC ground-truth region was 0.59-fold, indicating lower-than-area-proportional activation within the annotated diagnostic region. Warmer colors indicate regions of higher model activation.

**Figure S7. Representative images from the expert evaluation questionnaire.** In both representative comparisons, 100% of experts rated the more inclusive bounding box as better localized, as it encompassed a greater proportion of the diagnostically relevant structure; (**top**) Pseudohyphae (**bottom**) Epithelial-attached lactobacilli. These examples illustrate the experts’ consistent preference for bounding boxes that more completely capture key diagnostic features.

**Figure S8. workflow diagram summarizing the annotation process.** Archived teaching slides were used exclusively to construct the training dataset, whereas validation and test datasets were generated from a separate collection of prospectively collected vaginal discharge specimens from the Gynecology Outpatient Department of Maharaj Nakorn Chiang Mai Hospital. Vaginal discharge slides were processed by Gram or Giemsa staining as appropriate and examined by conventional light microscopy under oil immersion. Microscopic images were manually acquired using a smartphone positioned over the microscope eyepiece, with five representative fields of view collected per specimen. Images were curated and independently interpreted by a clinical microbiology expert. Diagnostic structures were annotated independently by at least two annotators, followed by expert review and adjudication of discrepancies to establish consensus ground-truth annotations. The finalized annotations were organized into training, validation, and test datasets with complete patient-level separation and prepared in YOLO-compatible format for model training.

**Figure S9. Confidence threshold sweep for YOLOv26 and RT-DETR across the test set (n=514).**
